# Epidemiological and Evolutionary Inference of the Transmission Network of the 2014 Highly Pathogenic Avian Influenza H5N2 Outbreak in British Columbia, Canada

**DOI:** 10.1038/srep30858

**Published:** 2016-08-04

**Authors:** Wanhong Xu, Yohannes Berhane, Caroline Dubé, Binhua Liang, John Pasick, Gary VanDomselaar, Soren Alexandersen

**Affiliations:** 1 Canadian Food Inspection Agency, National Centre for Foreign Animal Disease, Winnipeg, Manitoba, R3E 3M4, Canada; 2 Canadian Food Inspection Agency, Animal Health Risk Assessment, Ottawa, Ontario, K1A 0Y9, Canada; 3 Public Health Agency of Canada, National Microbiology Laboratory, Winnipeg, Manitoba, R3E 3M4, Canada; 4 Department of Biochemistry and Medical Genetics, University of Manitoba, Winnipeg, Manitoba, R3E 3R2, Canada; 5 Department of Medical Microbiology, University of Manitoba, Winnipeg, Manitoba, R3E 3R2, Canada; 6 Deakin University Geelong and Geelong Centre for Emerging Infectious Diseases, Geelong, Victoria 3220, Australia

## Abstract

The first North American outbreak of highly pathogenic avian influenza (HPAI) involving a virus of Eurasian A/goose/Guangdong/1/1996 (H5N1) lineage began in the Fraser Valley of British Columbia, Canada in late November 2014. A total of 11 commercial and 1 non-commercial (backyard) operations were infected before the outbreak was terminated. Control measures included movement restrictions that were placed on a total of 404 individual premises, 150 of which were located within a 3 km radius of an infected premise(s) (IP). A complete epidemiological investigation revealed that the source of this HPAI H5N2 virus for 4 of the commercial IPs and the single non-commercial IP likely involved indirect contact with wild birds. Three IPs were associated with the movement of birds or service providers and localized/environmental spread was suspected as the source of infection for the remaining 4 IPs. Viral phylogenies, as determined by Bayesian Inference and Maximum Likelihood methods, were used to validate the epidemiologically inferred transmission network. The phylogenetic clustering of concatenated viral genomes and the median-joining phylogenetic network of the viruses supported, for the most part, the transmission network that was inferred by the epidemiologic analysis.

Highly Pathogenic Avian Influenza (HPAI) which is caused by Influenza A viruses restricted to subtypes H5 and H7, is a highly contagious disease of poultry which, because of its transmissibility and associated high mortality rates, can have devastating consequences for the poultry industry^1^. For this reason, it is listed as a notifiable disease by the World Organization for Animal Health (OIE). Canada has an eradication strategy in place should HPAI viruses be detected in domestic poultry. Canada also has an ongoing serological surveillance program in the commercial poultry sector – the Canadian Notifiable Avian Influenza Surveillance System (CanNAISS), which is a joint initiative of government, industry and Canadian farmers to prevent, detect and/or demonstrate the freedom of notifiable avian influenza H5 and H7 subtypes in Canada’s domestic poultry flocks. In addition to surveillance in commercial poultry, annual surveys for influenza A viruses in wild birds, which began in 2005^2^,^3^, are used to assess the risk of exposure of poultry to H5 and H7 viruses occurring in migrating wild birds.

Prior to December 2014, Canada experienced two H7N3 HPAI outbreaks; one in British Columbia (BC) in 2004^4^ and a second in Saskatchewan in 2007^5^. Full genome sequencing on both of these viruses revealed that all 8 gene segments were of North American lineage. On December 1, 2014, a H5N2 HPAI virus was detected on 2 poultry farms in the Fraser Valley of BC; a chicken broiler breeder farm in Chilliwack and a meat turkey farm in Abbotsford. Full genome sequencing of the viruses isolated from both farms revealed them to be novel reassortants with PB2, PA, HA, M and NS genes related to a Eurasian H5N8 HPAI virus, and PB1, NP and NA genes related to North American lineage waterfowl viruses^6^.

During the months of October and November, tens of thousands of migrating waterfowl use the farm lands of the Fraser Valley as a resting stop before continuing their journey southward. Because of this, wild waterfowl were suspected as the most likely source of HPAI H5N2 infection for many of these farms. The fall migration period usually coincides with high rainfall (200–300 mm/month in October and November) which was the situation in 2014. In addition, the month of December recorded higher than normal average temperatures and rainfall. On many of the infected premises (IP), standing water occupied by large numbers of waterfowl was observed in the farm fields.

Over the course of the HPAI H5N2 outbreak a total of 11 commercial poultry farms (3 meat turkey, 1 table egg layer and 7 broiler breeder) and one non-commercial poultry or backyard operation were infected. A total of 240,000 animals were culled as part of the eradication strategy which included the depopulation of infected premises (IPs), tracing investigations, and the establishment of disease control zones (3 km infected zone and 10 km restricted zone) which included active surveillance as well as movement restrictions. The last HPAI H5N2 infected commercial farm was detected on December 17 and the infected non-commercial or backyard operation was detected on December 19, 2014. On February 2, 2015 a second non-commercial backyard operation infected with a novel reassortant HPAI H5N1 virus was identified. No other premises infected with this virus were identified following active surveillance. The last commercial farm to be released from quarantine was on March 25, 2015 after which post-outbreak surveillance commenced and was concluded on June 3, 2015.

Significant epidemiological investigations and data collection took place on each IP. Although suspected transmission networks were reasoned for 5 of the 11 commercial IPs, a clear source of infection could not be identified for the remaining 6 IPs. Therefore, the aim of this study was to integrate the genetic and epidemiological data from the 11 infected commercial poultry farms and the single non-commercial farm to gain a better understanding of the viral origin and transmission pathways and dynamics of this HPAI H5N2 outbreak.

## Results

### Epidemiological results

The first infected farm was detected on December 1, 2014 (IP1). This broiler breeder farm housed 8,000 hens and roosters aged 25 weeks in one barn and 4,800 hens and roosters aged 51 weeks in another barn. The first clinical sign noticed was an increase in mortality on November 28^th^. Also on December 1^st^ another infected farm (IP2) was detected. This turkey operation housed a total of 14,000 birds aged 2 weeks in one barn and 14,000 birds aged 12 weeks in two other barns. The first clinical sign was an increase in mortality that started on November 27^th^.

The epidemiological investigation did not reveal any links between IP1 and IP2 which suggested that wild birds were the most likely source of contamination for these farms. Notably, there were very heavy rains in late November, followed by freezing temperatures at the end of the month. Wild waterfowl and rodents seeking shelter in or around the barns may have contributed to the virus being introduced into the barns.

**Figure 1:**
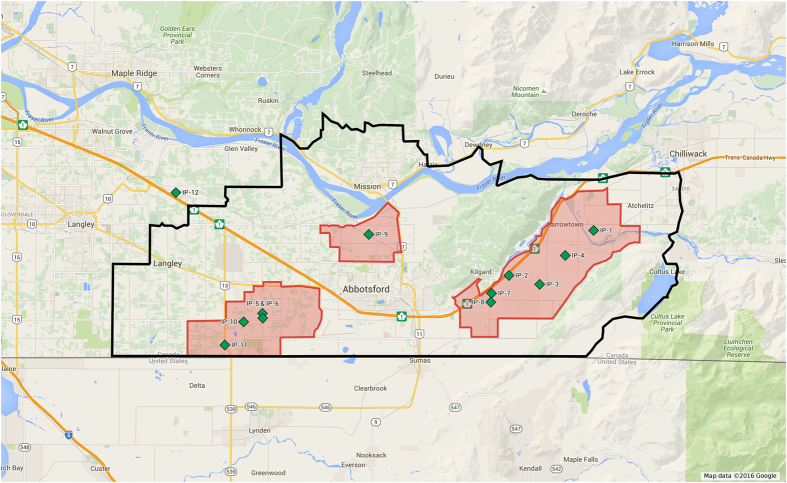
Spatial distribution of infected farms in the 2014 H5N2 outbreak in British Columbia, Canada. The green diamond shapes indicate the location of each infected premises designated IP1 to IP12. The black line indicates the boundary of the 10 km restricted zone and the red lines indicate the boundaries of the 3 km infected zones. The figure was produced utilizing Map data 2016^©^ Google complying with the Terms of Service as outlined at https://www.google.ca/permissions/geoguidelines.html with modifications.

Table 1 shows the timing of detection and reporting of clinical signs for each farm infected in the outbreak. Figure 1 shows the spatial distribution of the IPs in the Fraser Valley. The analysis of tracing data led to the identification of the following transmission network among 5 of the IPs:

1. IP1 to IP3 and IP4. IP1 shipped spiking males to IP4 on November 27^th^ and IP3 on November 28^th^. The first clinical signs on IP1 appeared on November 28^th^. The two recipient farms were placed under quarantine on December 2^nd^ when these movements were reported in epidemiological investigations (PIQs). Clinical signs were observed on December 1^st^ at IP4 and on December 3^rd^ at IP3 which suggests an incubation period of 4 to 5 days.
2. IP5 to IP6. IP5 was detected on December 4^th^. The two sites are owned by two brothers and actually represent two barns on a single site. Equipment sharing and low biosecurity between the two barns could have led to transmission from IP5 to IP6. Noteworthy is the fact that the two barns used the same catching crew on December 4^th^ (early morning after midnight). It caught birds at IP5 and then moved onto IP6 without practicing appropriate biosecurity measures. Close proximity and movement of people likely led to the transmission of the virus to IP6.

Careful study of movements of service providers and potential relationships among farms did not identify any other potential transmission events. Based on proximity and the timing of infection, it was hypothesized that IP2 transmitted infection to IP7 and IP8, through localized environmental spread/airborne spread. When exploring airborne spread as a potential secondary spread mechanism, only one farm was located within 1.5 km of IP2 and it did not become infected. Another four farms were located within 1.5 km–3 km of IP2: IP3, IP7, IP8 and a non-infected farm. No association was found between being located downwind from IP2 (cut-off for being considered exposed for 70% and 50% of the IP2’s contagious period) and becoming infected. Airborne spread was therefore ruled out. Other mechanisms of local/environmental spread were therefore suspected.

Investigations into potential sources of virus introduction into IP9 did not result in any links to other IPs. Here again, a point source introduction possibly resulting from indirect contact with wild waterfowl was considered the likely source of introduction of the virus.

Upon initial discussions with the owner of IP10, airborne spread was suspected. The producer reported the appearance of clinical signs in birds located just below an intake fan at the northeast end of the barn. The infection then spread from there to the other birds in the barn. The producer indicated that the intake fan of the northeast end of the barn was located downwind from IP5 and IP6 at the time of their depopulation. The depopulation procedures used in this outbreak required sealing the barns, closing the ventilation fans, injecting carbon dioxide (CO_2_) until the birds died and then turning the ventilation back on to evacuate the CO_2_. The venting process has been associated with the release of dust into the air and with proper meteorological conditions it is hypothesized that viral particles can be transported downwind and lead to infection of birds in the exposed farm^7^. The owner of IP10 practiced excellent biosecurity and had a number of measures in place to reduce the risk of introduction of HPAI. We therefore investigated the potential for airborne spread at the specific timeframe when depopulation took place at IP5 and IP6.

**Figure 2:**
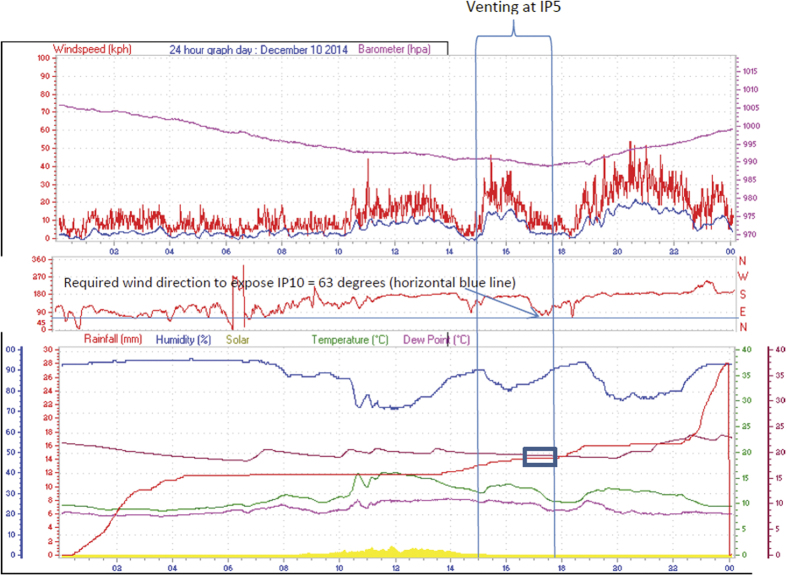
Rainfall, wind speed and wind direction recorded every 2 seconds at the local Abbotsford weather station on December 10^th^, 2014 (http://www.abbotsfordwx.com/wxhistory.php?date=201412). Wind speed is represented by a red line in the top graph, rainfall is represented by the red line in bottom graph and wind direction is represented by the red line in the center graph.

Depopulation took place on December 10^th^ at IP5 and December 11^th^ at IP6 and clinical signs first appeared on December 13^th^ at IP10. The venting process occurred at 15:30–18:15 on December 10^th^. Meteorological conditions during that time period (Fig. 2) indicated that there was no precipitation (16:45–18:00). During this time period the wind direction varied 67.5–135 degrees (ENE to SE). IP10 is located at a 63 degree angle and 1.5 km from IP5. Airborne spread was therefore considered as a possible spread mechanism from IP5 to IP10. In the case of IP6 as a potential source, the venting process took place at 17:15–18:15 on December 11^th^. There was no precipitation reported during that time period and the wind direction ranged between 90–135 degrees. IP 10 was located at a 70 degree angle and 1.5 km from IP6 and considering the very short incubation this would represent (2 days), it was considered less likely that depopulation events at IP6 led to infection of IP10.

In the case of IP11, it was considered to be part of the cluster of infected farms that included IPs 5, 6 and 10. Since it was not located within 3 km of IP5 or IP6, it was not considered at risk of airborne spread following depopulation at these locations. Also, the birds on IP11 developed clinical signs on December 19^th^, which would have represented a long incubation period (8–9 days) for airborne spread transmission. IP11 is located within 3 km of IP10 and airborne spread from IP10 was also explored. There were four farms located within 3 km of IP10, 2 exposed (including IP11) and 2 non-exposed. Airborne spread was considered not to be a statistically significant mechanism of transmission and as a result, it was concluded that some form of localized/environmental spread was responsible for infection of IP11.

**Figure 3:**
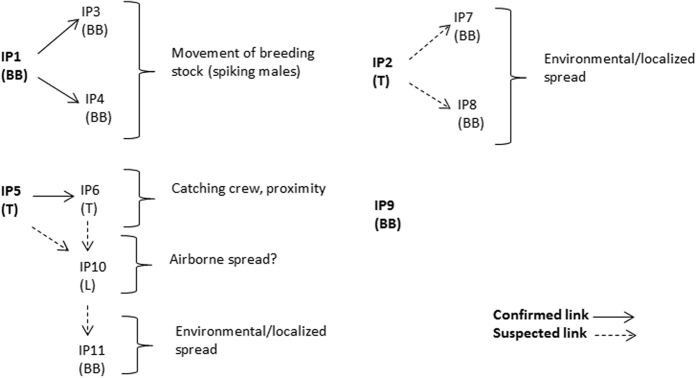
Proposed transmission network of the H5N2 outbreak in British Columbia, 2014, resulting from the field epidemiological investigation and analysis. IP: infected premises, BB: broiler breeder, L: layer, T: turkey.

Figure 3 shows the hypothesized transmission network based on the analysis of field data recorded in the PIQ and the additional investigations carried out by the field epidemiologists.

### Origin and evolutionary rate of HPAI H5N2 viruses

**Figure 4:**
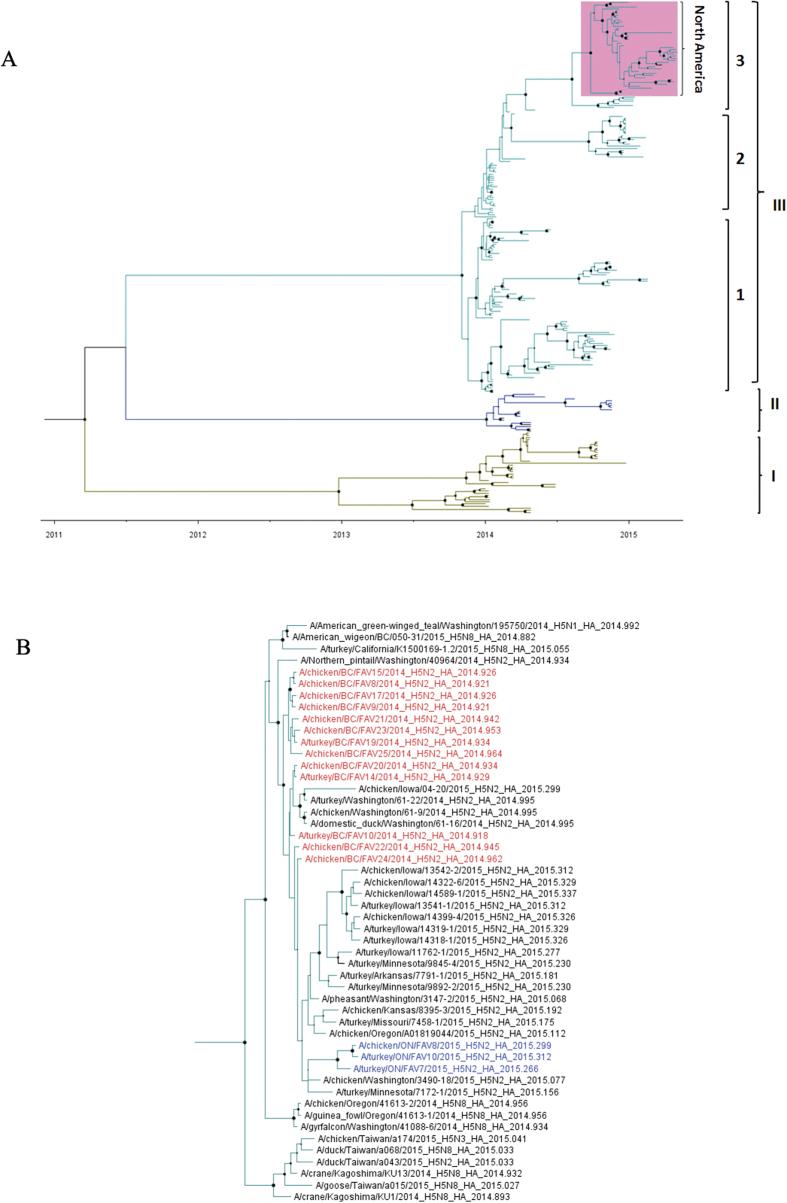
(**A**) Time-scaled phylogenetic tree inferred using Bayesian MCMC analysis for HA gene of H5-clade 2.3.4.4 viruses. Nodes and branches are coloured according to clusters formed. North America H5-clade 2.3.4.4 viruses are highlighted in magenta. Nodes are indicated by the solid circles and sized by posterior probability. Fully annotated tree is available in Supplemental Figure 1. (**B**) Close-up of cluster III (3) from A with name of viruses labeled. British Columbia (BC) H5N2 outbreak viruses are shown in red and Ontario H5N2 outbreak viruses in blue.

We inferred the evolutionary relationships of H5 sequences of 239 H5 clade 2.3.4.4 viruses collected between January 1, 2014 and July 10, 2015 (Fig. 4, Figure S1). The analysis showed that the outbreak H5N2 HA sequences are most similar to the HA sequences from Asian H5N8 viruses, which is consistent with previous conclusions that the H5N2 outbreak strains were the result of a reassortment event^6^,^8^,^9^. Rates of nucleotide substitution and time of the most recent common ancestor (TMRCA) of the BC HPAI H5N2 viruses were then estimated for each gene segment separately as well as for the eight concatenated segments using a Bayesian Markov Chain Monte Carlo (BMCMC) method implemented in BEAST^10^. The analyses showed that the mean substitution rates were high for the HA gene (12.14 × 10^−3^ substitutions/site/year), the NA gene (9.10 × 10^−3^ substitutions/site/year) and the NS gene (11.39 × 10^−3^ substitutions/site/year) (Table 2). The estimated rates for the other genome segments were lower, ranging from 4.51 × 10^−3^ substitutions/site/year for the PB2 gene segment to 7.44 × 10^−3^ substitutions/site/year for the PB1 gene segment. The substitution rates in this study are similar to those reported for H7N1 and H7N7^11^ viruses. TMRCA estimations indicated that the origin of the BC HPAI H5N2 viruses dated back to between mid-June 2014 (mean) for the HA gene to late August 2014 for NA gene (mean; Table 2), suggesting that the reassortant event may have occurred more than 4 months before the initial outbreak was detected on December 1, 2014.

### Virus transmission pathways based on phylogenetic analysis

**Figure 5:**
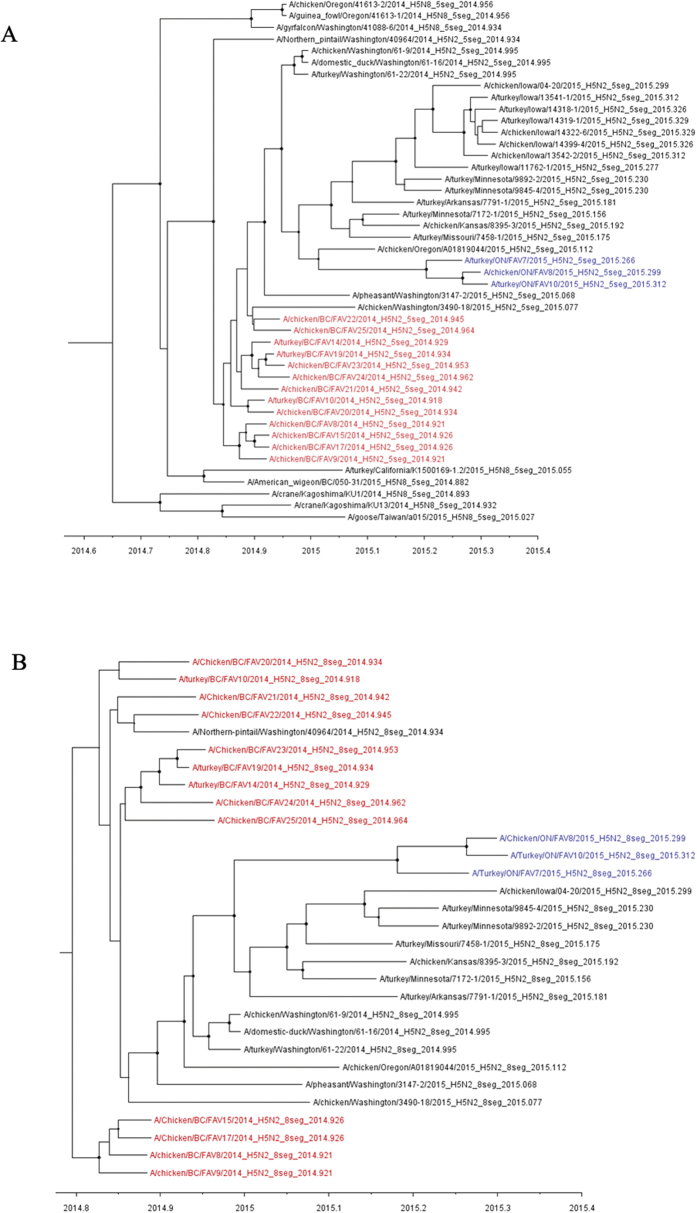
(**A**) MCC tree inferred for the five concatenated Eurasian origin gene segments (PB2, PA, HA, M, and NS) of the North America H5-clade 2.3.4.4 viruses containing H5N8 and H5N2 subtypes. The closest Eurasian strains inferred from supplemental Figure S2 are included in the analyses. (**B**) MCC tree inferred for the eight concatenated gene segments of North America H5N2 viruses of H5-clade 2.3.4.4. In both A and B, nodes are indicated by the solid circles and sized by posterior probability. The Canadian H5N2 outbreak viruses are coloured in the same manner as shown in (**B**).

Prior to carrying out phylogenetic analysis on the five and eight concatenated gene sequence data sets the presence of reassortants were screened for using the RPD4 software package. No reassortants were identified in the dataset involving the 30 members of the eight concatenated gene dataset. However, reassortant sequences were identified in the initial five concatenated gene dataset which was comprised of 129 sequences. These sequences, derived from viruses A/duck/Taiwan/a043/2015 (H5N2), A/duck/Taiwan/a068/2015 (H5N8), and A/broiler duck/Korea/H49/2014 (H5N8) were excluded from subsequent phylogenetic analysis. Based on the phylogenetic analyses of the five concatenated genes of Eurasian (PB2, PA, HA, M and NS) lineage (Fig. 5A), and the eight concatenated genes of Eurasian (PB2, PA, HA, M and NS) and North American (PB1, NP and NA) lineage (Fig. 5B), viruses from the following infected premises (IP) were shown to cluster together: IP1 (BC/FAV8 and BC/FAV9) clusters with IP3 (BC/FAV15) and IP4 (BC/FAV17); IP2 (BC/FAV10) clusters with IP7 (BC/FAV20); IP5 (BC/FAV14) clusters with IP6 (BC/FAV19) and IP10 (BC/FAV23) and to a lesser extent IP11 (BC/FAV24); and IP8 (BC/FAV21), IP9 (BC/FAV22) and IP12 (BC/FAV25) all appear separate and not to cluster with any of the above. This time-scaled phylogenetic clustering is in general agreement with the conclusions made based on the epidemiologic investigation in that at least four independent incursions of HPAI H5N2 took place corresponding to IP1, IP2, IP5 and IP9. Based on phylogenetic analysis, IP 8 and the backyard operation IP12 also appear to be separate incursions. All of the aforementioned IPs are located many kilometers apart with no known epidemiological link. The phylogenetic clustering inferred by Bayesian MCMC analysis was confirmed by maximum likelihood analysis using the HKY + G substitution model and 1000 bootstrap replicates (Figures S3–S5).

**Figure 6:**
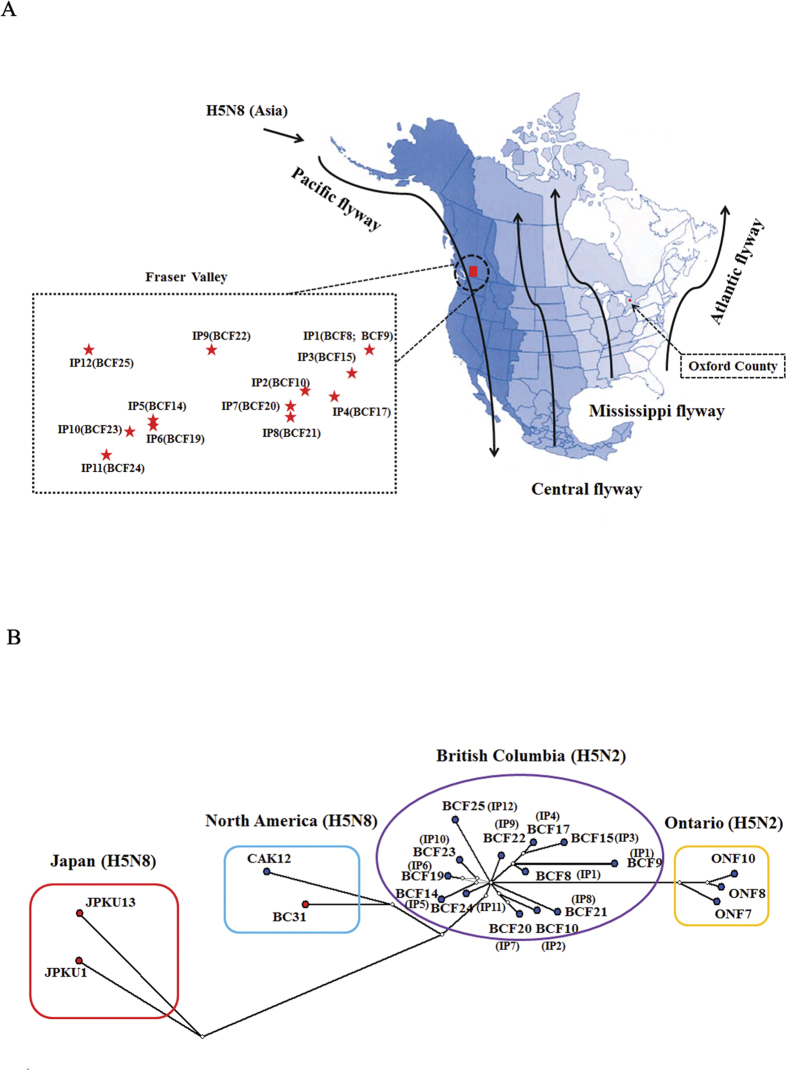
(**A**) Geographic map showing North America migratory waterfowl flyways (Adapted from Nelson and Bartonek^9^ (1990) with modifications and permission from Wildlife Management Institute, Gardners, Pennsylvania). The four flyways are represented by gradient shades of blue. Locations of Canadian H5N2 outbreaks are indicated by red on the map. The relative locations of BC H5N2 index premises (IP) in the area of Fraser Valley are shown in the dotted box. The representative sample collected at each IP was shown in the parentheses beside the name of IP. (**B**) Median-joining phylogenetic network of H5-clade 2.3.4.4 viruses of Canadian H5N2 outbreak viruses in relation to H5N8 viruses. The median-joining network was constructed from the concatenated PB2, PA, HA, M, and NS sequence data. This network includes all of the most parsimonious trees linking the sequences. Each unique sequence is represented by a circle showing the same frequency in the dataset. Branch length is proportional to the number of mutations. Viruses isolated from poultry are represented by blue dots while viruses isolated from wild birds are represented by red dots.

Four flyways exist in North America and the regions covered by each flyway are shown in Fig. 6A (adapted from Nelson and Bartonek)^12^. The BC HPAI H5N2 outbreak viruses were the result of a reassortant event involving an Asian origin H5N8 and a North American low-pathogenicity AIV (LPAIV) that most likely took place within the Pacific flyway^6^,^8^,^9^. To assess the inter-farm transmission network, the concatenated sequences from HA, PA, M, PB2, and NS were used to construct a Median Joining phylogenetic network (Fig. 6B). The network included all maximum parsimony trees, thus representing all the plausible evolutionary pathways linking the farm samples. Figure 6B shows that BC HPAI H5N2 sequences are distinct compared to Ontario HPAI H5N2, North American H5N8, and Asian H5N8 sequences supporting the multiple clusters of infection identified by epidemiologic investigations. Sequences within clusters were separated on average by 4–7 nucleotide differences, whereas 13–16 nucleotide differences were observed between clusters. We did not identify farm samples that shared identical sequences and there were no unambiguous connections found in the phylogenetic network (i.e. BC HPAI H5N2 sequences were derived from one or more calculated ancestors and were not descendants of each other).

### Amino acid substitutions of HPAI H5N2

Phylogenetic analysis of the five concatenated gene segments PB2, PA, HA, M and NS showed that all of the BC HPAI H5N2 viruses most likely evolved from a virus related to A/Northern pintail/Washington/40964/2014 (GenBank accession numbers KP307973.1, KP307975.1, KP307976.1, KP307979.1 and KP307980.1) (Fig. 5A). Thus, A/Northern pintail/Washington/40964/2014 was used as a reference strain to infer amino acid substitutions of the BC HPAI H5N2 outbreak viruses. Amino acid substitutions were observed in all gene segments (Table 3). Out of a total 59 substitutions, singleton sites were defined as follows: 8 in PB2, 6 in PB1, 5 in PA, 4 in HA, 5 in NP, 9 in NA, 4 in NS1, and 2 in NS2. Although the biological functions of these substitutions are largely unknown, the introduction of a valine at position 105 of NP has been shown to be critical for high pathogenicity^13^.

All of the BC HPAI H5N2 viruses contain a number of amino acid substitutions that have previously been associated with recognized phenotypic traits that include host range, virulence and antiviral drug resistance. These include: residues 123 P, 133 A, 156A, and 235P in HA (based on the numbering system suggested by Burke and Smith^14^) which have been associated with increased binding to α-2,6-linked glycans; residue 66S in PB1-F2 associated with increased replication efficiency and weight loss in mice^15^; residues 30D and 215A in M1 and residues 42S, 103F and 106M in NS1 associated with increased virulence and systemic infection; residues 227-230ESEV in NS1 associated with increased weight loss in mice; residue 701D in PB2 associated with systemic infection; and residue 31N in M2 associated with decreased sensitivity to amantadine and/or rimantadine.

### Selection pressures acting on the viral genes

The selection pressures acting on each viral gene segment were assessed as described in Materials and Methods and the results summarized in Table 4. The analysis revealed that the vast majority of amino acid sites were affected by purifying selection. Only the REL method identified residue 215 (posterior probability, 0.999) in PB1 and residues 105 (posterior probability, 0.999) and 109 (posterior probability, 0.967) in NP as being under positive selection. It is worth noting that both of these genes are of North American lineage, and that along with PB2 and PA, are involved in viral RNA replication and transcription. Whether these changes represent a transient polymorphism or adaptive evolution to optimize protein-protein interaction and function remains to be determined. Residue 215 in PB1 is located within the nuclear localization motif (amino acid positions 203–216)^16^. Residues 105 and 109 in NP are part of the RNA binding sub-region (amino acid positions 79–180)^17^. Valine at amino acid position 105 in NP has been reported as one of the determinants for adaptation of avian influenza virus from ducks to chickens^13^. This substitution was only found in chicken samples from IP 11. The high evolutionary rate observed for the NS gene segment (11.39 × 10^−3^ substitutions/site/year) (Table 2) along with a mean dN-dS difference of 0.288 (Table 4), indicating positive selection is noteworthy. The NS1 protein is a non-essential virulence factor that has multiple accessory functions that include inhibition of host innate immune responses^18^. The above values arrived at for evolutionary rate and selection pressure may also indicate adaptive evolution to a new gene constellation or host.

## Discussion

Reconstructing transmissions trees of outbreaks of infectious disease have been approached in a number of ways. These have been based on the analysis of detailed epidemiologic data, the analysis of the amount of genetic diversity observed in the pathogen from different cases involved in an outbreak, and the integration of epidemiologic and genetic data^19^,^20^,^21^,^22^. With respect to avian influenza outbreaks, Bataille *et al*.^19^ have shown that virus evolution over the course of an outbreak is rapid enough to permit the use of genetic data to reconstruct inter-farm transmission. The approaches used to integrate epidemiologic and genetic data to generate transmission trees have varied. Cottam *et al*.^20^ used genetic data to exclude a subset of potential transmission trees and then evaluated the remaining transmission trees with the epidemiologic data to come up with a small number of transmission trees that have the highest likelihood of supporting the epidemiologic data. Jombart *et al*.^21^ followed a similar approach using an algorithm called SeqTrack which directly reconstructs the most plausible genealogy of a set of sampled isolates, enabling the direct assessment of the epidemic’s spatiotemporal dynamics. Ypma *et al*.^22^ used a far more sophisticated approach that combines the genetic and epidemiologic data into one likelihood function which is then used in a Bayesian setting to sample from the space of all transmission trees. The current study took a relatively simple approach comparing the transmission network deduced from the epidemiologic, geographic and historical weather data with that inferred from the phylogenies of the viruses isolated from each IP.

Our analysis showed that the viral phylogenies based on the five concatenated Eurasian origin gene segments were essentially the same as the phylogenies based on all eight concatenated gene segments. Importantly, phylogenetic support for the clustering and transmission pathways elucidated from the epidemiologic investigations can only be considered reliable if the assumption is made that the virus genotype obtained for each IP is the dominant strain. Deep sequencing carried out on samples collected from each IP (data not shown) reinforces the notion that this was the case.

One of the associations explored in this study was whether being downwind from an infected farm correlated with becoming infected. Only a weak epidemiological association indicating possible airborne spread was found to exist between IP5 and IP10 or IP6 and IP10, although genetic analysis did demonstrate a strong association among these three IPs. Although airborne spread may have played a role in other avian influenza outbreaks, we concluded that it did not appear to be an important mechanism of spread for the 2014 British Columbia HPAI H5N2 outbreak. Broilers, which were not affected in this outbreak, were excluded from the analysis. The reasoning for this decision was based on the premise that if no association between being located downwind from an infected farm and becoming infected was found with broilers excluded, then including broilers in the analysis would not have changed our final conclusions. Had an epidemiological association between being located downwind from an infected farm and becoming infected been found, including broilers in the analysis would likely have rendered that association insignificant due to their large numbers and the fact that none were infected in this outbreak. Why broilers were not affected during this outbreak is not known but may be due to a number of factors that might include differences in susceptibility, biosecurity or levels of stress.

**Figure 7:**
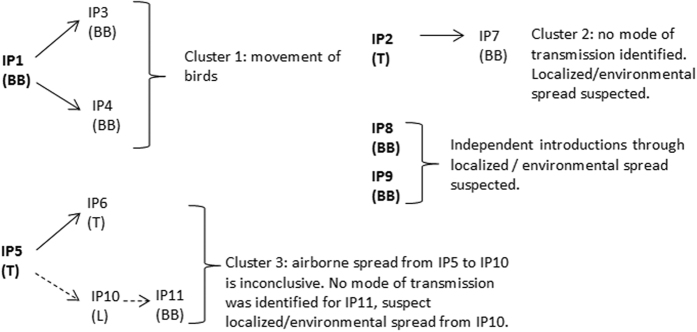
Final transmission network of the HPAI H5N2 outbreak in British Columbia, 2014, combining epidemiological and genetic data analysis. IP: infected premises, BB: broiler breeder, L: layer, T: turkey.

Many of the hypotheses generated by analysis of the epidemiologic data were supported by the genetic analyses. Point source introductions, identified by field epidemiologic analysis and which likely resulted from indirect contact with wild waterfowl, were supported for IPs 1, 2, 5, 9 and the non-commercial or backyard IP 12. Genetic analysis also corroborated linkages between IP 1 and IPs 3 and 4 as well as those between IPs 5 and 6. The suspected link between IPs 2 and 7 was bolstered by the genetic analysis and since no clear mode of transmission was identified, local spread/environmental spread is hypothesized as the most likely mechanism. However, links between IP2 and IP8 were not supported by the phylogenetic analysis indicating that IP8 may be another point source introduction through indirect contact with wild waterfowl. A similar conclusion, based on phylogenetic analysis, was also arrived at for IP9. Results of genetic analysis supported the strong epidemiological association that existed between IP5 and IP6 as well as the link grounded on the proximity, with IP10. Based on the geographic proximity between IP5/IP6 and IP10 (1.5 km), the likelihood of airborne spread between either IP5 and IP10 or IP6 and IP10 was investigated. Although airborne spread between IP5 and IP10 as based on the weather conditions that were recorded at the time of barn venting following CO_2_ euthanasia remains a possibility, this mechanism should still be considered inconclusive and it is more likely that some other local/environmental means of spread was responsible. Finally, IP11 was indeed part of the cluster of IP5, 6 and 10, although a clear mode of transmission was not established and therefore local/environmental spread is also suspected. The final transmission network combining field epidemiology and phylogenetic analysis proposes three clusters of infection and is shown in Fig. 7.

High resolution genetic analysis of infectious disease agents involved in outbreaks is proving extremely useful in confirming or disproving hypotheses inferred from field epidemiologic data. In this particular study, links among farms were confirmed but the actual mechanisms responsible for linking those farms were not identified for the majority of the IPs. In many of the IPs, even though the biosecurity was good it did not prevent entry of the virus into the barns suggesting a mechanism of introduction that may not easily lend itself to control. In other instances trucks that had visited an infected farm did not spread the infection to as many as 9–10 other farms that they subsequently visited, suggesting that either the biosecurity was very good in these subsequent farms or the virus was not very contagious and required a specific set of conditions to initiate an infection. Studies similar to the one described here will incrementally add to our understanding of the parameters that affect virus transmission dynamics.

## Materials and Methods

All experiments carried out on live vertebrates associated with this study were carried out in accordance with guidelines set out by the Canadian Council on Animal Care and all experimental manipulations carried out on live animals were approved by the Canadian Science Centre for Human & Animal Health Animal Care Committee.

### Epidemiological investigation

The Premises Investigation Questionnaire (PIQ) was administered on each infected farm. The PIQ (see supplementary information) collects information on flock demographics and management, on the history of clinical signs and on contacts that may have taken place on and off the farm. Information on the presence of wild waterfowl, standing water in surrounding fields and rodent control were also obtained in the PIQ in order to guide the identification of potential mechanisms of virus introduction.

The information related to on and off contacts was then investigated further to determine potential sources of infection for the infected farm and potential further dispersal of the virus to other farms. In the case of direct contacts (movement of birds), the recipient or source farms were placed under quarantine and investigated for clinical signs. In the case of indirect contacts, which include visits by service providers such as feed deliveries, egg collectors or bird catchers, their logs were obtained for a period of 72 hours prior to and following the visit on an infected farm. In the case of movement of people or sharing of equipment, these were investigated on a case-by-case basis to determine the level of risk the contact represented and quarantines were implemented if the risk was judged to be high.

**Figure 8:**
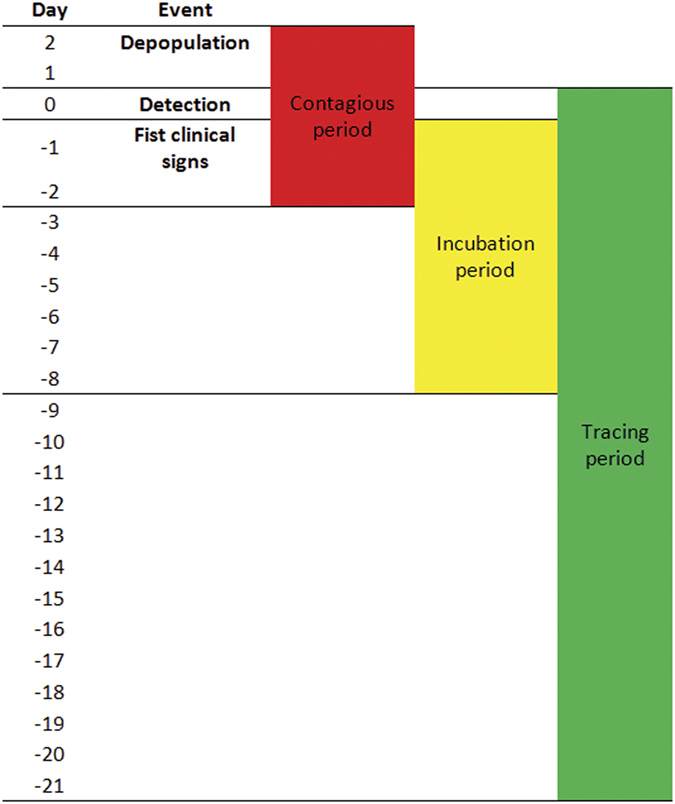
Time periods used for all epidemiological investigations on each infected farm in the 2014 H5N2 outbreak in British Columbia, Canada.

For each infected farm, the following important dates and timeframes (see Fig. 8) were calculated: 1) the tracing critical period of 21 days prior to the date of appearance of clinical signs, 2) the incubation period calculated as 7 days prior to the reported date of appearance of clinical signs and 3) the contagious period calculated as one day prior to the date of appearance of clinical signs until the date of depopulation. All contacts of an infected farm during the contagious period were investigated to determine if these contacts could have led to secondary spread. If the recipient of a contact developed clinical signs following an incubation period of 3–7 days, secondary spread was considered to have taken place. All contacts onto an infected farm +/−2 days before or after the start of the incubation period were investigated to determine if these contacts could have led to the introduction of the virus into the infected farm. If the source farm was infected and contagious at the time of the contact, it was identified as the origin of the virus for the infected farm of concern.

When no contacts could explain the introduction of the virus onto an infected farm, other mechanisms of spread were investigated. These included the presence of wild waterfowl in or around the infected barn, water wells and water treatment, rodent control, the potential for airborne spread and other environmental/localized spread mechanisms.

Airborne spread has been suspected as a potential spread mechanism for HPAI in recent outbreaks^7^,^23^,^24^. High volume air samples collected downwind from infected farms in the 2004 HPAI outbreak in British Columbia led to the identification of the presence of viral genomic material at 800 m from an infected farm^7^,^24^. In the 2015 HPAI outbreak in the United States, genetic material was detected at a distance of 70 m to 1,000 m. A dispersion modelling study concluded that the odds of becoming infected significantly increased for farms located within 5 km of an infected farm^25^.

In the present study, each infected farm was thought to excrete virus at high levels throughout its contagious period (one day prior to the appearance of clinical signs until depopulation). All commercial poultry farms with the exception of chicken broiler farms, located within 3 km of the infected farm were identified. Their degree location relative to each infected farm was calculated as described below. A “Map distance and direction 1 km Non-Broilers” spreadsheet was generated using the ArcGIS “Generate Near Table” geoprocessing tool utilizing the most centrally located sub-premises on each IP as the input features layer and all non-IP sub-premises in BC as the near features layer. Both feature layers were saved in the Universal Transverse Mercator (UTM) projected coordinate system to ensure the distance results were calculated in meters. The “Generate Near Table” geoprocessing tool produced a table listing distance and direction from all input features to all near features. This table was queried to identify the closest sub-premises on each premise to the most centrally located sub-premises on each IP. Values beyond 3 km were removed, and the remaining distance and direction values were recorded in the spreadsheet. For information on the geographic methodology of the ArcGIS “Generate Near Table” geoprocessing tool, please refer to http://resources.arcgis.com/EN/HELP/MAIN/10.2/index.html#/How_proximity _tools_calculate_distance/000800000048000000/.

In assessing the potential of airborne spread, chicken broiler farms were excluded from the analysis as they were not affected in this outbreak. Adding these types of birds would have further diluted the potential association between being located downwind from an IP and becoming infected.

Meteorological data obtained every 2 seconds at a local weather station in Abbotsford was obtained for each day of an IP’s contagious period (http://abbotsfordwx.com/index.php). Total wind direction range covered during the day when there was an absence of precipitation was recorded (heavy precipitation will lead to deposition of particles to the ground). A farm was considered exposed if it was downwind from an IP for at least 70% of an IP’s contagious period (a 50% cutoff was also used for comparison). A resulting two-by-two table was created to explore the association between being located downwind from an IP and becoming exposed. The Fisher’s exact test was used to explore the association with a p-value <0.05 considered as significant.

When airborne spread was suspected at a certain point in time (at the time of depopulation of an IP for example), similar weather data was obtained for the time period of concern and a qualitative risk assessment of the potential spread downwind was performed.

### Nucleotide sequences used in the study

AIV sequences were downloaded from the GISAID database under virus collection date starting from 1 January 2014 to the date of downloading (i.e. 10 July 2015). Only H5 clade 2.3.4.4 viruses were selected for phylogenetic analyses (Table S1). The nucleotides in the coding regions of segments 1 (PB2), 2 (PB1), 3 (PA), 4 (HA), 5 (NP), and 6 (NA) were aligned using ClustalW^26^ followed by manual adjustment to codon position. The full nucleotide sequences of segments 7 (M1 and M2) and 8 (NS1 and NS2) were also aligned using ClustalW, and the sequences were edited such that all of the codons in first open reading frame (ORF) were followed by the remaining codons in the second ORF (i.e. nucleotides were not repeated between the two ORFs) in MEGA v6.06^27^. A total of 239 HA (H5) sequences were used to construct a time-scaled phylogenetic tree (Figure S1). Of the selected H5 clade 2.3.4.4 viruses, nucleotides that encode PB2, PA, HA, M1, M2, NS1, and NS2 of 126 strains containing H5N8 or H5N2 subtype were manually concatenated for analyses (Figure S2). Manual concatenation of the eight segments for 30 North American HPAI H5N2 viruses that included the viruses associated with this outbreak was also done.

### Phylogenetic analyses and transmission network construction

Rates of nucleotide substitution per site per year and the time to the most recent common ancestor (TMRCA) of Canadian HPAI H5N2 viruses were estimated using the Bayesian Markov Chain Monte Carlo (MCMC) method^10^ as implemented in the program BEAST, version 2.3.0^28^. Due to limited sampling timespan of the viruses, we used a simpler model to avoid over-parameterization. In this regard a single Hasegawa–Kishino–Yano (HKY) model that best fit the different datasets with gamma-distributed rates among sites was used^29^. The age of the viruses was defined as the date of sample collection. Coalescent constant population was used for tree prior and a strict clock model was selected for analyses. For each dataset, at least two independent runs were conducted for 50 million generations, sampling every 5000 generations. Convergences and effective sample sizes (ESS) of the estimates were checked using Tracer v1.6^30^. All parameter estimates for each run showed ESS values > 400. A maximum clade credibility (MCC) phylogenetic tree was generated to summarize all 10,000 trees after a 10% burn-in using TreeAnnotator in BEAST^28^. All time-stamped phylogenetic trees were visualized and annotated using FigTree v1.4.2^31^. The topologies of the MCC trees were compared to those inferred using the maximum likelihood (ML) method in the PhyML program^32^.

The concatenated five gene segments that encode the seven proteins of Eurasian lineage (PB2, PA, HA, M1, M2, NS1, and NS2) were used to construct a phylogenetic network using the Median Joining method implemented in the program Network v4.6.1.3 (http://fluxus-engineering.com/). The parameter epsilon, which controls the level of homoplasy, was set at the same value as the weight of characters used to calculate the genetic distances (weight value = 10). A similar analysis was carried out using the concatenated eight gene segments for 30 North American HPAI H5N2 viruses that included the viruses that were the focus of this study.

Maximum likelihood phylogenetic analyses, using MEGA 6.06 software^27^, was also carried out on the three datasets (i.e. 30 concatenated 8 gene segments of North American H5N2, 45 concatenated 5 gene segments of North American H5N2 and H5N8, and 239 H5 gene segments) in order to compare with the Bayesian method. For this, the best fit nucleotide substitution model, HKY + G was used. To assess the robustness of different nodes, bootstrap analysis was undertaken using 1000 replicates of the data set.

### Analysis of selection pressure

Site-specific selection pressures for all segments of the North America H5N2 viruses were measured as nonsynonymous (dN) -synonymous (dS) nucleotide substitutions per site. In all cases, the difference were estimated using the single-likelihood ancestor counting (SLAC), fixed-effects likelihood (FEL), internal fixed-effects likelihood (IFEL), and random effects likelihood (REL) methods^33^,^34^ available at the Datamonkey^35^,^36^ online version of the HyPhy package^37^. All analyses utilized the HKY85 nucleotide substitution model, which was tested as the best fitting model for the data sets, and employed input Neighbor Joining phylogenetic trees. A cut-off p-value to classify a site as positively or negatively selected was set at 0.01 for SLAC, FEL, and IFEL methods. The cut-off value for the Bayes factor in the REL method was set at 100 to reflect a positive or negative selection at a given site.

### Screening for recombinants and reassortants among the outbreak viruses

Multiple sequence alignments were screened for recombinant and reassortant sequences using the programs RDP, GENECONV, MAXCHI, CHIMAERA, 3SEQ, BOOTSCAN and SISCAN as implemented in the RDP4 software package^38^ using default settings. Potential recombinant/reassortant sequences were identified when two or more methods were in agreement with p-values <0.001. No recombinants were identified in the dataset involving 239 HA sequences and no reassortants were identified among the 30 members of the eight concatenated gene dataset. Reassortant sequences were however identified in the five concatenated gene dataset which was initially comprised of 129 sequences. These sequences which were derived from viruses A/duck/Taiwan/a043/2015 (H5N2), A/duck/Taiwan/a068/2015 (H5N8), and A/broiler duck/Korea/H49/2014 (H5N8) were excluded from subsequent phylogenetic analysis.

## Additional Information

**How to cite this article**: Xu, W. *et al*. Epidemiological and Evolutionary Inference of the Transmission Network of the 2014 Highly Pathogenic Avian Influenza H5N2 Outbreak in British Columbia, Canada. *Sci. Rep.* **6**, 30858; doi: 10.1038/srep30858 (2016).

## Supplementary Material

Supplementary Information

Supplementary Information

## Figures and Tables

**Table 1:** Description of each infected farm in the H5N2 outbreak in British Columbia, Canada, 2014.

IP#	Submission#	Type of birds	^#^Birds in affected barn	First clinical signs	Detection (H5)^1^	Destruction Completed
1	FAV8 and FAV9	Broiler breeder	13,000	Nov. 28	Dec. 1	Dec. 5
2	FAV10	Turkey	28,000	Nov. 27	Dec. 1	Dec. 6
3	FAV15	Broiler breeder	14,000	Dec. 3	Dec. 3	Dec. 7
4	FAV17	Broiler breeder	27,000	Dec. 1	Dec. 2	Dec. 8
5	FAV14	Turkey	30,000	Dec. 4	Dec. 6	Dec. 10
6	FAV19	Turkey	30,000	Dec. 7	Dec. 9	Dec. 11
7	FAV20	Broiler breeder	18,000	Dec. 8	Dec. 10	Dec. 13
8	FAV21	Broiler breeder	9,000	Dec. 9	Dec. 10	Dec. 13
9	FAV22	Broiler breeder	6,000	Dec. 9	Dec. 10	Dec. 14
10	FAV23	Table Egg Layer	53,000	Dec. 13	Dec. 13	Dec. 16
11	FAV24	Broiler breeder	12,000	Dec. 17	Dec. 17	Dec. 19
12	FAV25	Non-commercial	85	Dec. 17	Dec. 19	Dec. 20

^1^Represents the day when non-negative H5 PCR test result were obtained.

**Table 2:** Estimated rates of nucleotide substitution and TMRCA of H5N2 outbreaks in British Columbia and Ontario Canada, 2014 and 2015.

Gene	Substitution rate (x 10^−3^)		TMRCA^b^ (day/month/year)	
	Mean	95% HPD^a^	Mean	95% HPD
HA	12.14	5.34–20.00	12/06/2014	26/05/2014–07/07/2014
NS	11.39	2.93–22.10	23/07/2014	29/05/2014–23/10/2014
NA	9.1	2.41–16.80	22/08/2014	08/06/2014–15/12/2014
PB1	7.44	2.22–13.60	08/07/2014	28/05/2014–09/09/2014
PA	6.91	2.17–12.00	05/07/2014	30/05/2014–31/08/2014
NP	5.53	0.99–10.70	31/07/2014	28/05/2014–27/11/2014
M	4.92	0.64–10.50	27/07/2014	26/05/2014–04/12/2014
PB2	4.51	1.30–8.17	16/07/2014	30/05/2014–27/09/2014

^a^95% highest posterior density intervals.

^b^time of most recent common ancestor.

**Table 3:** Non-synonymous mutations detected within the protein coding regions compared to A/Northern-pintail/Washington/40964/2014 (H5N2).

Strain	Protein								
	PB2	PB1	PA	HA^b^	NP	NA	M2	NS1	NS2
A/chicken/BC/FAV8/2014 (H5N2)	L386V			L8F, N130T, N416S		R338W, R435M, E368K			
A/chicken/BC/FAV9/2014 (H5N2)	M47I, L386V, K721E			L8F, N130T, N416S	S3P	L35S, R338W, R435M, E368K			
A/turkey/BC/FAV10/2014 (H5N2)	L386V	L373I		L8F, N130T		E368K			
A/turkey/BC/FAV14/2014 (H5N2)	L386V, S534P		G152R	L8F, N130T	I109T^a^	E368K		D237G	I80V
A/chicken/BC/FAV15/2014 (H5N2)	L386V	M414V	V669I	L8F, N130T, N416S	T15I	R338W, R435M, E368K			R86K
A/chicken/BC/FAV17/2014 (H5N2)	L386V			L8F, N130T, N416S		R338W, R435M, E368K			R86K
A/turkey/BC/FAV19/2014 (H5N2)	L386V, L435P, S534P			L8F, N130T	I109T^a^	E368K			
A/chicken/BC/FAV20/2014 (H5N2)	L386V	L298P		L8F, N130T		E368K, I469V			
A/chicken/BC/FAV21/2014 (H5N2)	L386V	M111T		L8F, N130T, F257L		H274R, E368K		R224K	E67K
A/chicken/BC/FAV22/2014 (H5N2)	L386V		V700I	L8F, N130T		E368K			
A/chicken/BC/FAV23/2014 (H5N2)	L386V, S534P			L8F, N130T, D171N	I109T^a^	E368K		V136M	
A/chicken/BC/FAV24/2014 (H5N2)	L386V, S534P		G58D, F672L	L8F, N130T	M105V^a^, I257T	P46L, E368K			
A/chicken/BC/FAV25/2014 (H5N2)	M66I, L386V			L8F, N130T, I178V	Q327L	I305V, D309G, S332Y, E368K			
A/turkey/ON/FAV7/2015 (H5N2)	L386V, T530A, I559T, V649I	R215K^a^		I4T, L8F, N130T, V226M		R253K, V412A, N463D, E368K	Q78R	I176T	
A/chicken/ON/FAV8/2015 (H5N2)	T97A, L386V, V649I		N321K	I4T, L8F, N130T, V226M		R253K, V412A, N463D, E368K	Q78R	I176T	
A/turkey/ON/FAV10/2015 (H5N2)	L386V, V649I	T25I	N321K	I4T, L8F, N130T, V226M		E83G, R253K, V412A, N463D, E368K	Q78R	T127P, I176T	

^a^Positively selected amino acid changes.

^b^Amino acid numbering based on the hemagglutinin precursor.

**Table 4:** Amino acid sites under putative positive selection.

Gene	Mean dN-dS^a^	Amino acid site^b^	Posterior probability
PB2	−0.676		
PB1	−0.803	215	0.999
PA	−0.770		
HA	−0.669		
NP	−0.586	105	0.999
		109	0.967
NA	−0.427		
M1	−1.000		
M2	3.159		
NS1	0.288		
NS2	−0.514		

^a^Values were calculated by using random effects likelihood (REL) method for all genes.

^b^Positively selected sites were determined by using REL method.
