# Supplementary material for: Epidemiological and Evolutionary Inference of the Transmission Network of the 2014 Highly Pathogenic Avian Influenza H5N2 Outbreak in British Columbia, Canada
Source: Sci Rep. 2016 Aug 4;6:30858. doi: 10.1038/srep30858 (PMC4973242; doi:10.1038/srep30858)
Supplement: Supplementary Information [file srep30858-s2.pdf]

Supplementary Material

Epidemiological and Evolutionary Inference of the Transmission Network of the 2014 Highly Pathogenic Avian Influenza H5N2 Outbreak in British Columbia, Canada

Wanhong Xu[1], Yohannes Berhane[1], Caroline Dubé[2], Binhua Liang[3,5], John Pasick[1*], Gary VanDomselaar[3,6], Soren Alexandersen[1,4]

Figure S1. MCC tree inferred for HA gene sequences of clade 2.3.4.4 H5 viruses collected between January 1, 2004 and July 10, 2015. The olive green coloured branches represent cluster I, the blue coloured branches represent cluster II, and the teal coloured branches represent cluster III. Nodes are indicated by the solid circles and sized by posterior probability.

Figure S2. MCC tree inferred for five concatenated Eurasian origin gene segments (PB2, PA, HA, M, and NS) of H5-clade 2.3.4.4 viruses collected between January 1, 2004 and July 10, 2015. Only H5N8 and H5N2 subtypes are included in the analyses. Nodes are indicated by the solid circles and sized by posterior probability.

Figure S3. ML tree of the HA gene sequences of clade 2.3.4.4 H5 viruses collected between January 1, 2004 and July 10, 2015. The numbers at nodes represent bootstrap values (>70%). Branch lengths are scaled according to the numbers of nucleotide substitutions per site.

Figure S4. ML tree of the five concatenated Eurasian origin gene segments (PB2, PA, HA, M, and NS) of the North American H5 clade 2.3.4.4 viruses containing H5N8 and H5N2 subtypes. The numbers at nodes represent bootstrap values (>70%). Branch lengths are scaled according to the numbers of nucleotide substitutions per site.

Figure S5. ML tree of the eight concatenated gene segments of the North American HPAI H5N2 viruses. The numbers at nodes represent bootstrap values (>70%). Branch lengths are scaled according to the numbers of nucleotide substitutions per site.

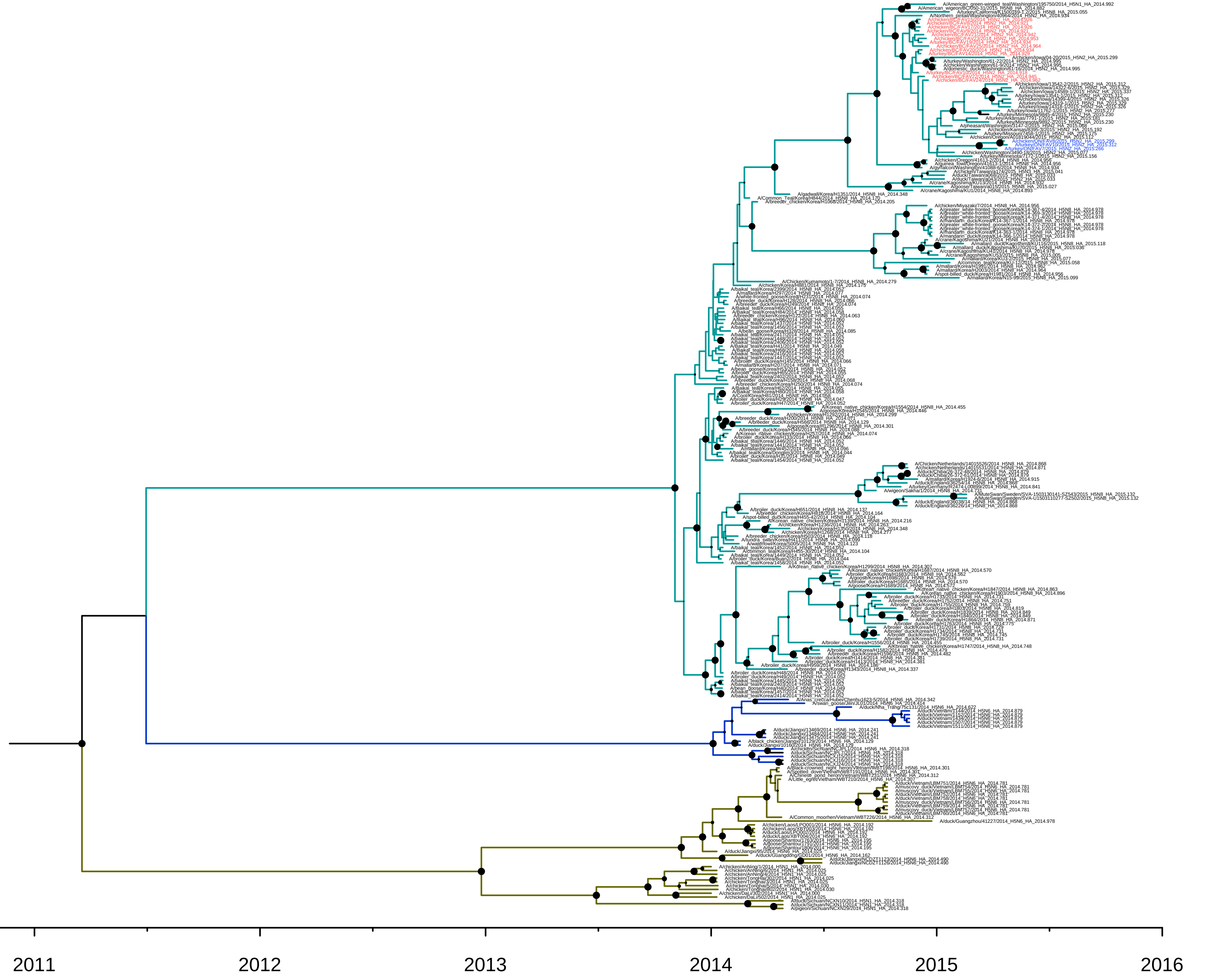
2011
2012
2013
2014
2015
2016

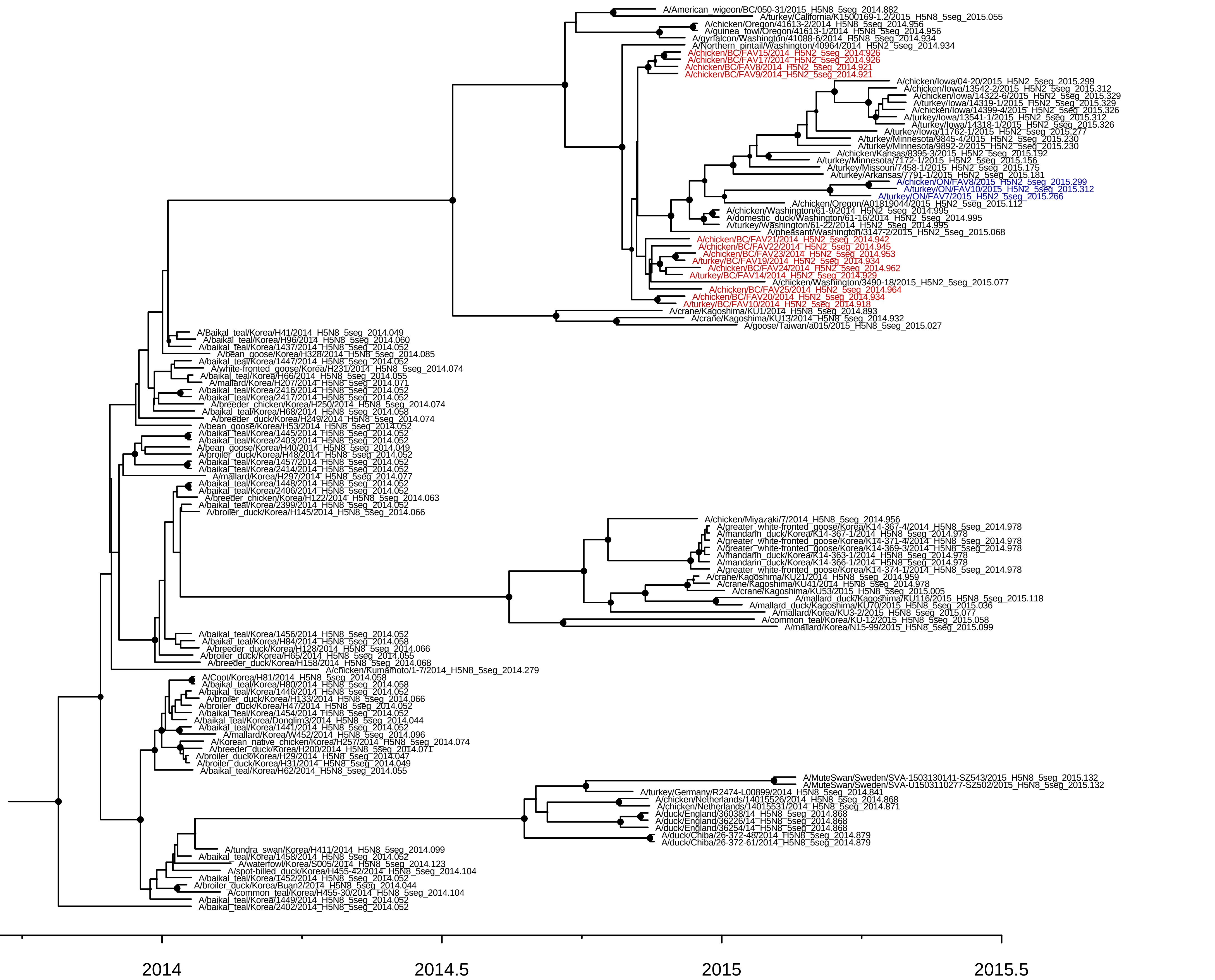

A/American_wigeon/BC/050-31/2015_H5N8_5seg_2014.882
A/turkey/California/K1500169-1.2/2015_H5N8_5seg_2015.055
A/chicken/Oregon/41613-2/2014_H5N8_5seg_2014.956
A/guinea_fowl/Oregon/41613-1/2014_H5N8_5seg_2014.956
A/gyrfalcon/Washington/41088-6/2014_H5N8_5seg_2014.934
A/Northern_pintail/Washington/40964/2014_H5N2_5seg_2014.934
A/chicken/BC/FAV15/2014_H5N2_5seg_2014.926
A/chicken/BC/FAV17/2014_H5N2_5seg_2014.926
A/chicken/BC/FAV8/2014_H5N2_5seg_2014.921
A/chicken/BC/FAV9/2014_H5N2_5seg_2014.921
A/chicken/Iowa/04-20/2015_H5N2_5seg_2015.299
A/chicken/Iowa/13542-2/2015_H5N2_5seg_2015.312
A/chicken/Iowa/14322-6/2015_H5N2_5seg_2015.329
A/turkey/Iowa/14319-1/2015_H5N2_5seg_2015.329
A/chicken/Iowa/14399-4/2015_H5N2_5seg_2015.326
A/turkey/Iowa/13541-1/2015_H5N2_5seg_2015.312
A/turkey/Iowa/14318-1/2015_H5N2_5seg_2015.326
A/turkey/Iowa/11762-1/2015_H5N2_5seg_2015.277
A/turkey/Minnesota/9845-4/2015_H5N2_5seg_2015.230
A/turkey/Minnesota/9892-2/2015_H5N2_5seg_2015.230
A/chicken/Kansas/8395-3/2015_H5N2_5seg_2015.192
A/turkey/Minnesota/7172-1/2015_H5N2_5seg_2015.156
A/turkey/Missouri/7458-1/2015_H5N2_5seg_2015.175
A/turkey/Arkansas/7791-1/2015_H5N2_5seg_2015.181
A/chicken/ON/FAV8/2015_H5N2_5seg_2015.299
A/turkey/ON/FAV10/2015_H5N2_5seg_2015.312
A/turkey/ON/FAV7/2015_H5N2_5seg_2015.266
A/chicken/Oregon/A01819044/2015_H5N2_5seg_2015.112
A/chicken/Washington/61-9/2014_H5N2_5seg_2014.995
A/domestic_duck/Washington/61-16/2014_H5N2_5seg_2014.995
A/turkey/Washington/61-22/2014_H5N2_5seg_2014.995
A/pheasant/Washington/3147-2/2015_H5N2_5seg_2015.068
A/chicken/BC/FAV21/2014_H5N2_5seg_2014.942
A/chicken/BC/FAV22/2014_H5N2_5seg_2014.945
A/chicken/BC/FAV23/2014_H5N2_5seg_2014.953
A/turkey/BC/FAV19/2014_H5N2_5seg_2014.934
A/chicken/BC/FAV24/2014_H5N2_5seg_2014.962
A/turkey/BC/FAV14/2014_H5N2_5seg_2014.929
A/chicken/Washington/3490-18/2015_H5N2_5seg_2015.077
A/chicken/BC/FAV25/2014_H5N2_5seg_2014.964
A/chicken/BC/FAV20/2014_H5N2_5seg_2014.934
A/turkey/BC/FAV10/2014_H5N2_5seg_2014.918
A/crane/Kagoshima/KU1/2014_H5N8_5seg_2014.893
A/crane/Kagoshima/KU13/2014_H5N8_5seg_2014.932
A/goose/Taiwan/a015/2015_H5N8_5seg_2015.027
A/Baikal_teal/Korea/H41/2014_H5N8_5seg_2014.049
A/baikal_teal/Korea/H96/2014_H5N8_5seg_2014.060
A/baikal_teal/Korea/1437/2014_H5N8_5seg_2014.052
A/bean_goose/Korea/H328/2014_H5N8_5seg_2014.085
A/baikal_teal/Korea/1447/2014_H5N8_5seg_2014.052
A/white-fronted_goose/Korea/H231/2014_H5N8_5seg_2014.074
A/baikal_teal/Korea/H66/2014_H5N8_5seg_2014.055
A/mallard/Korea/H207/2014_H5N8_5seg_2014.071
A/baikal_teal/Korea/2416/2014_H5N8_5seg_2014.052
A/baikal_teal/Korea/2417/2014_H5N8_5seg_2014.052
A/breeder_chicken/Korea/H250/2014_H5N8_5seg_2014.074
A/baikal_teal/Korea/H68/2014_H5N8_5seg_2014.058
A/breeder_duck/Korea/H249/2014_H5N8_5seg_2014.074
A/bean_goose/Korea/H53/2014_H5N8_5seg_2014.052
A/baikal_teal/Korea/1445/2014_H5N8_5seg_2014.052
A/baikal_teal/Korea/2403/2014_H5N8_5seg_2014.052
A/bean_goose/Korea/H40/2014_H5N8_5seg_2014.049
A/broiler_duck/Korea/H48/2014_H5N8_5seg_2014.052
A/baikal_teal/Korea/1457/2014_H5N8_5seg_2014.052
A/baikal_teal/Korea/2414/2014_H5N8_5seg_2014.052
A/mallard/Korea/H297/2014_H5N8_5seg_2014.077
A/baikal_teal/Korea/1448/2014_H5N8_5seg_2014.052
A/baikal_teal/Korea/2406/2014_H5N8_5seg_2014.052
A/breeder_chicken/Korea/H122/2014_H5N8_5seg_2014.063
A/baikal_teal/Korea/2399/2014_H5N8_5seg_2014.052
A/broiler_duck/Korea/H145/2014_H5N8_5seg_2014.066
A/chicken/Miyazaki/7/2014_H5N8_5seg_2014.956
A/greater_white-fronted_goose/Korea/K14-367-4/2014_H5N8_5seg_2014.978
A/mandarin_duck/Korea/K14-367-1/2014_H5N8_5seg_2014.978
A/greater_white-fronted_goose/Korea/K14-371-4/2014_H5N8_5seg_2014.978
A/greater_white-fronted_goose/Korea/K14-369-3/2014_H5N8_5seg_2014.978
A/mandarin_duck/Korea/K14-363-1/2014_H5N8_5seg_2014.978
A/mandarin_duck/Korea/K14-366-1/2014_H5N8_5seg_2014.978
A/greater_white-fronted_goose/Korea/K14-374-1/2014_H5N8_5seg_2014.978
A/crane/Kagoshima/KU21/2014_H5N8_5seg_2014.959
A/crane/Kagoshima/KU41/2014_H5N8_5seg_2014.978
A/crane/Kagoshima/KU53/2015_H5N8_5seg_2015.005
A/mallard_duck/Kagoshima/KU116/2015_H5N8_5seg_2015.118
A/mallard_duck/Kagoshima/KU70/2015_H5N8_5seg_2015.036
A/mallard/Korea/KU3-2/2015_H5N8_5seg_2015.077
A/common_teal/Korea/KU-12/2015_H5N8_5seg_2015.058
A/mallard/Korea/N15-99/2015_H5N8_5seg_2015.099
A/baikal_teal/Korea/1456/2014_H5N8_5seg_2014.052
A/baikal_teal/Korea/H84/2014_H5N8_5seg_2014.058
A/breeder_duck/Korea/H128/2014_H5N8_5seg_2014.066
A/broiler_duck/Korea/H65/2014_H5N8_5seg_2014.055
A/breeder_duck/Korea/H158/2014_H5N8_5seg_2014.068
A/chicken/Kumamoto/1-7/2014_H5N8_5seg_2014.279
A/Coot/Korea/H81/2014_H5N8_5seg_2014.058
A/baikal_teal/Korea/H80/2014_H5N8_5seg_2014.058
A/baikal_teal/Korea/1446/2014_H5N8_5seg_2014.052
A/broiler_duck/Korea/H133/2014_H5N8_5seg_2014.066
A/broiler_duck/Korea/H47/2014_H5N8_5seg_2014.052
A/baikal_teal/Korea/1454/2014_H5N8_5seg_2014.052
A/baikal_teal/Korea/Donglim3/2014_H5N8_5seg_2014.044
A/baikal_teal/Korea/1441/2014_H5N8_5seg_2014.052
A/mallard/Korea/W452/2014_H5N8_5seg_2014.096
A/Korean_native_chicken/Korea/H257/2014_H5N8_5seg_2014.074
A/breeder_duck/Korea/H200/2014_H5N8_5seg_2014.071
A/broiler_duck/Korea/H29/2014_H5N8_5seg_2014.047
A/broiler_duck/Korea/H31/2014_H5N8_5seg_2014.049
A/baikal_teal/Korea/H62/2014_H5N8_5seg_2014.055
A/MuteSwan/Sweden/SVA-1503130141-SZ543/2015_H5N8_5seg_2015.132
A/MuteSwan/Sweden/SVA-U1503110277-SZ502/2015_H5N8_5seg_2015.132
A/turkey/Germany/R2474-L00899/2014_H5N8_5seg_2014.841
A/chicken/Netherlands/14015526/2014_H5N8_5seg_2014.868
A/chicken/Netherlands/14015531/2014_H5N8_5seg_2014.871
A/duck/England/36038/14_H5N8_5seg_2014.868
A/duck/England/36226/14_H5N8_5seg_2014.868
A/duck/England/36254/14_H5N8_5seg_2014.868
A/duck/Chiba/26-372-48/2014_H5N8_5seg_2014.879
A/duck/Chiba/26-372-61/2014_H5N8_5seg_2014.879
A/tundra_swan/Korea/H411/2014_H5N8_5seg_2014.099
A/baikal_teal/Korea/1458/2014_H5N8_5seg_2014.052
A/waterfowl/Korea/S005/2014_H5N8_5seg_2014.123
A/spot-billed_duck/Korea/H455-42/2014_H5N8_5seg_2014.104
A/baikal_teal/Korea/1452/2014_H5N8_5seg_2014.052
A/broiler_duck/Korea/Buan2/2014_H5N8_5seg_2014.044
A/common_teal/Korea/H455-30/2014_H5N8_5seg_2014.104
A/baikal_teal/Korea/1449/2014_H5N8_5seg_2014.052
A/baikal_teal/Korea/2402/2014_H5N8_5seg_2014.052
2014
2014.5
2015
2015.5

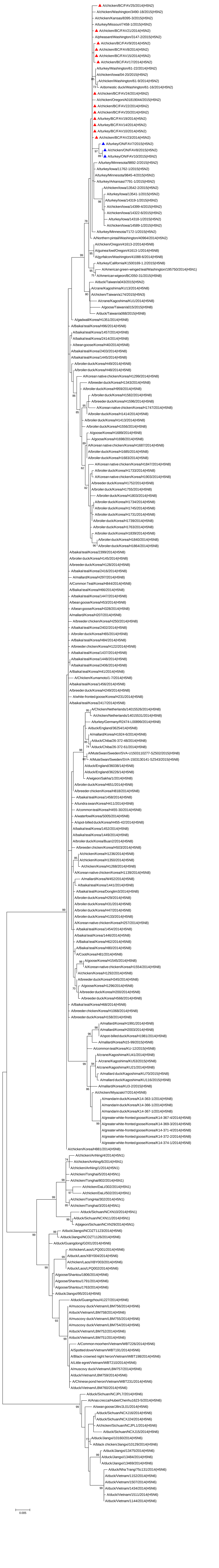

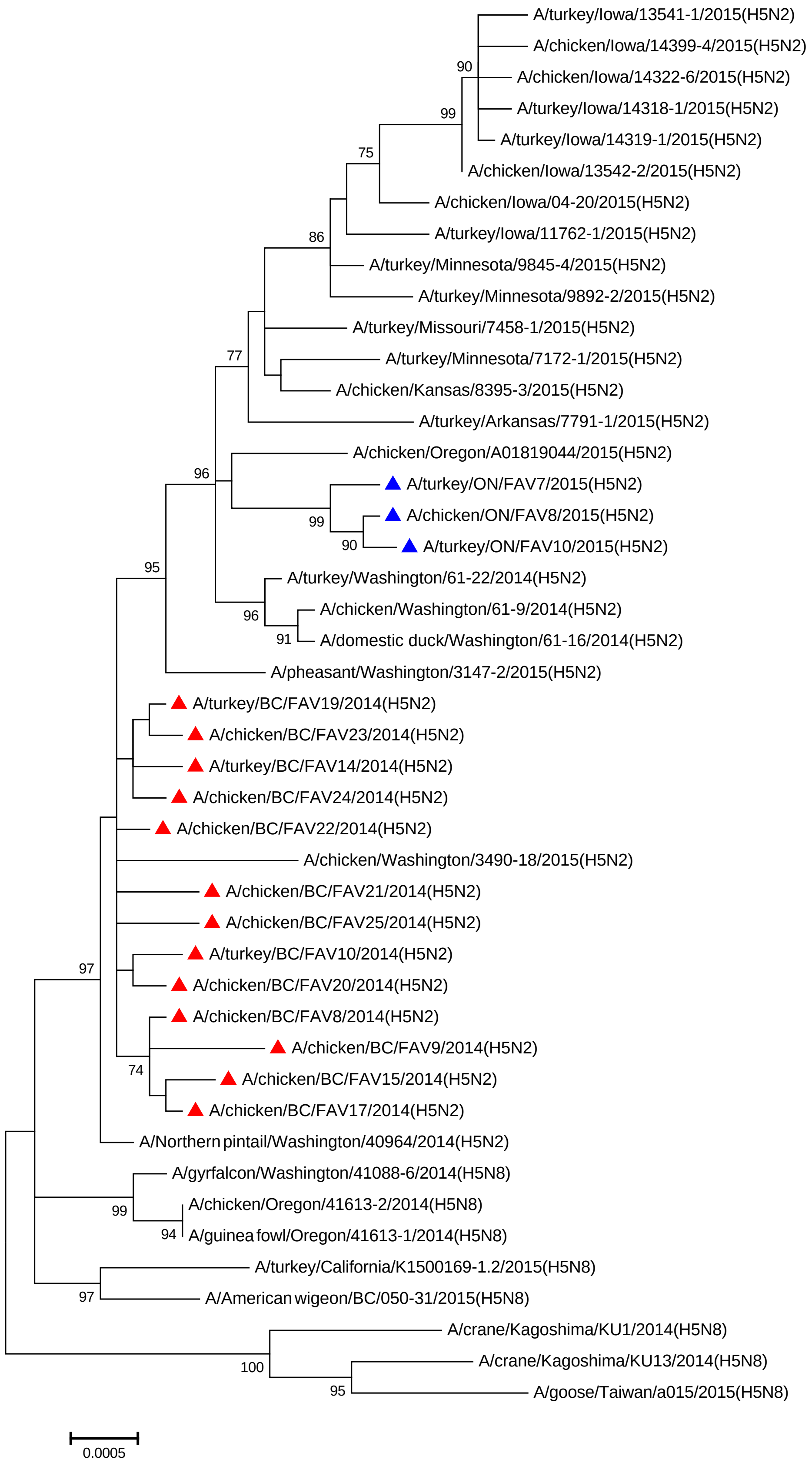

A/turkey/Iowa/13541-1/2015(H5N2)
A/chicken/Iowa/14399-4/2015(H5N2)
A/chicken/Iowa/14322-6/2015(H5N2)
A/turkey/Iowa/14318-1/2015(H5N2)
A/turkey/Iowa/14319-1/2015(H5N2)
A/chicken/Iowa/13542-2/2015(H5N2)
A/chicken/Iowa/04-20/2015(H5N2)
A/turkey/Iowa/11762-1/2015(H5N2)
A/turkey/Minnesota/9845-4/2015(H5N2)
A/turkey/Minnesota/9892-2/2015(H5N2)
A/turkey/Missouri/7458-1/2015(H5N2)
A/turkey/Minnesota/7172-1/2015(H5N2)
A/chicken/Kansas/8395-3/2015(H5N2)
A/turkey/Arkansas/7791-1/2015(H5N2)
A/chicken/Oregon/A01819044/2015(H5N2)
A/turkey/ON/FAV7/2015(H5N2)
A/chicken/ON/FAV8/2015(H5N2)
A/turkey/ON/FAV10/2015(H5N2)
A/turkey/Washington/61-22/2014(H5N2)
A/chicken/Washington/61-9/2014(H5N2)
A/domestic duck/Washington/61-16/2014(H5N2)
A/pheasant/Washington/3147-2/2015(H5N2)
A/turkey/BC/FAV19/2014(H5N2)
A/chicken/BC/FAV23/2014(H5N2)
A/turkey/BC/FAV14/2014(H5N2)
A/chicken/BC/FAV24/2014(H5N2)
A/chicken/BC/FAV22/2014(H5N2)
A/chicken/Washington/3490-18/2015(H5N2)
A/chicken/BC/FAV21/2014(H5N2)
A/chicken/BC/FAV25/2014(H5N2)
A/turkey/BC/FAV10/2014(H5N2)
A/chicken/BC/FAV20/2014(H5N2)
A/chicken/BC/FAV8/2014(H5N2)
A/chicken/BC/FAV9/2014(H5N2)
A/chicken/BC/FAV15/2014(H5N2)
A/chicken/BC/FAV17/2014(H5N2)
A/Northern pintail/Washington/40964/2014(H5N2)
A/gyrfalcon/Washington/41088-6/2014(H5N8)
A/chicken/Oregon/41613-2/2014(H5N8)
A/guinea fowl/Oregon/41613-1/2014(H5N8)
A/turkey/California/K1500169-1.2/2015(H5N8)
A/American wigeon/BC/050-31/2015(H5N8)
A/crane/Kagoshima/KU1/2014(H5N8)
A/crane/Kagoshima/KU13/2014(H5N8)
A/goose/Taiwan/a015/2015(H5N8)
90
99
75
86
77
96
99
90
95
96
91
97
74
99
94
97
100
95
0.0005

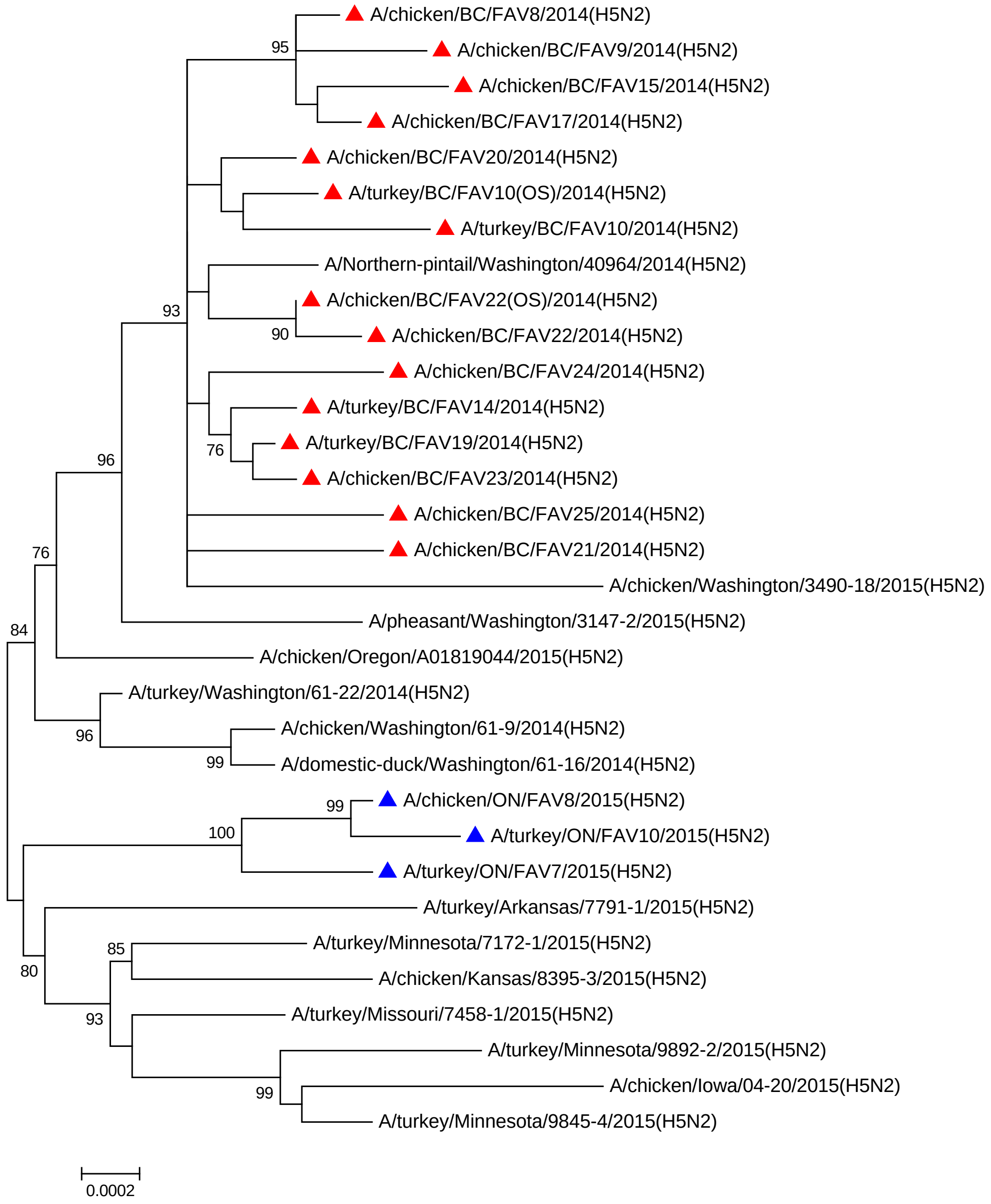

A/chicken/BC/FAV8/2014(H5N2)
A/chicken/BC/FAV9/2014(H5N2)
A/chicken/BC/FAV15/2014(H5N2)
A/chicken/BC/FAV17/2014(H5N2)
A/chicken/BC/FAV20/2014(H5N2)
A/turkey/BC/FAV10(OS)/2014(H5N2)
A/turkey/BC/FAV10/2014(H5N2)
A/Northern-pintail/Washington/40964/2014(H5N2)
A/chicken/BC/FAV22(OS)/2014(H5N2)
A/chicken/BC/FAV22/2014(H5N2)
A/chicken/BC/FAV24/2014(H5N2)
A/turkey/BC/FAV14/2014(H5N2)
A/turkey/BC/FAV19/2014(H5N2)
A/chicken/BC/FAV23/2014(H5N2)
A/chicken/BC/FAV25/2014(H5N2)
A/chicken/BC/FAV21/2014(H5N2)
A/chicken/Washington/3490-18/2015(H5N2)
A/pheasant/Washington/3147-2/2015(H5N2)
A/chicken/Oregon/A01819044/2015(H5N2)
A/turkey/Washington/61-22/2014(H5N2)
A/chicken/Washington/61-9/2014(H5N2)
A/domestic-duck/Washington/61-16/2014(H5N2)
A/chicken/ON/FAV8/2015(H5N2)
A/turkey/ON/FAV10/2015(H5N2)
A/turkey/ON/FAV7/2015(H5N2)
A/turkey/Arkansas/7791-1/2015(H5N2)
A/turkey/Minnesota/7172-1/2015(H5N2)
A/chicken/Kansas/8395-3/2015(H5N2)
A/turkey/Missouri/7458-1/2015(H5N2)
A/turkey/Minnesota/9892-2/2015(H5N2)
A/chicken/Iowa/04-20/2015(H5N2)
A/turkey/Minnesota/9845-4/2015(H5N2)
95
93
90
76
96
76
84
96
99
99
100
80
85
93
99
0.0002

PART B1: INITIAL PREMISES VISIT REPORT - INITIAL ASSESSMENT AND SAMPLING

INVESTIGATION ID: ____________________

Premises Identifier / Name: ____________________

B1.1 Date of arrival (YY/MM/DD): _____ / _____ / _____ Time of arrival (24 hr):

Licence plates of CFIA vehicles used for visit:

B1.2 GPS coordinate of main entrance way: (units are decimal degrees, to 5 decimal places)

LAT: **LONG: -**

B1.3 Name and title of person(s) accompanying CFIA staff on the premises visit:

B1.4 Provide a complete description of the location of this premises (i.e. street address, mailing address, legal land description, etc.):

<u>Confirm</u> contact numbers for the premises:

Phone: Cellular: Fax:

B1.5 **<u>Confirm</u>** CONTACT PERSON for the premises:

Address:

Title(s):

<u>Confirm</u> contact numbers:

Phone: Cellular: Fax:

B1.6 **<u>Confirm</u>** OWNER of the premises:

Address:

<u>Confirm</u> contact numbers for the OWNER of the premises:

Phone: Cellular: Fax:

B1.7 **<u>Confirm</u>** OWNER(S) of the animals on site:

Address:

<u>Confirm</u> contact numbers for the OWNER(S) of the animals:

Phone: Cellular: Fax:

PART B1: INITIAL PREMISES VISIT REPORT - INITIAL ASSESSMENT AND SAMPLING

INVESTIGATION ID: ______________________

Premises Identifier / Name: ______________________

B1.8 List ANY OTHER PEOPLE connected to the animals on this premises in the table below:

NAME	ADDRESS	PHONE	HOW ARE THEY CONNECTED?

B1.9 ANIMAL PRODUCTION TYPE

(1) How would you characterize this operation / premises? (Check ALL that are appropriate.)

- ☐ ARTIFICIAL INSEMINATION UNIT
- ☐ BREEDING - OUTDOOR RANGE
- ☐ COMMERCIAL - OUTDOOR RANGE
- ☐ COMMERCIAL - TOTAL CONFINEMENT BARN
- ☐ COMMERCIAL - SEMI-CONFINEMENT BARN
- ☐ EDUCATIONAL - SCHOOL
- ☐ EDUCATIONAL - ZOO, PUBLIC ACCESS
- ☐ EXHIBITION / FAIRGROUND / COMPETITION FACILITY
- ☐ HUNT PRESERVE - OUTDOOR RANGE
- ☐ HUNT PRESERVE - SUPPLIER
- ☐ LABORATORY / RESEARCH FACILITY
- ☐ NON-COMMERCIAL - OUTDOOR RANGE
- ☐ NON-COMMERCIAL - SEMI-CONFINEMENT BARN
- ☐ NON-COMMERCIAL - TOTAL CONFINEMENT BARN
- ☐ NUCLEUS OR MULTIPLIER BREEDER HERD
- ☐ PARENT / GRANDPARENT STOCK (AVIAN)
- ☐ PET STORE / SALES FACILITY
- ☐ PUREBRED - OUTDOOR RANGE
- ☐ PUREBRED - SEMI-CONFINEMENT BARN
- ☐ PUREBRED - TOTAL CONFINEMENT BARN
- ☐ QUARANTINE STATION / EXPORT ASSEMBLY
- ☐ VELVET PRODUCTION
- ☐ VETERINARY HOSPITAL / CLINIC
- ☐ OTHER - Describe:

(2) Is this premises part of a multi-site production unit? **YES** _____ / **NO** _____

If YES: Please describe:

(3) This premises is a ‘closed’ production unit (i.e. ‘all-in / all-out’ by premises). **YES** _____ / **NO** _____

If NO: Please describe:

PART B1: INITIAL PREMISES VISIT REPORT - INITIAL ASSESSMENT AND SAMPLING

INVESTIGATION ID: ______________________

Premises Identifier / Name: ______________________

B1.9 ANIMAL PRODUCTION TYPE (continued)

(4) Check off ALL types of animals on this premises.
Identify which of these are the MAIN types (i.e. financial importance) to this operation.

AVIAN:	Present on Premises?	Identify (√) MAIN type(s)
Chicken - meat type - broiler	☐	________
Chicken - meat type - broiler breeder	☐	________
Chicken - meat type - broiler breeder pullets	☐	________
Chicken - meat type - broiler breeder roosters	☐	________
Chicken - egg type - layer	☐	________
Chicken - egg type - pullet	☐	________
Chicken - egg type - breeder	☐	________
Chicken - egg type - breeder pullets	☐	________
Chicken - dual purpose	☐	________
Emu	☐	________
Gamebird - pheasant	☐	________
Gamebird - quail	☐	________
Gamebird - other: ______________________	☐	________
Import - illegal - type: ____________________	☐	________
Import - legal - type: _____________________	☐	________
Ostrich	☐	________
Pigeon - racing	☐	________
Pigeon - squab	☐	________
Raptor	☐	________
Rhea	☐	________
Specialty - type: _________________________	☐	________
Specialty breeder - type: __________________	☐	________
Tinamou	☐	________
Turkey - breeder	☐	________
Turkey - meat	☐	________
Turkey - replacement breeder	☐	________
Waterfowl - duck	☐	________
Waterfowl - duck breeder	☐	________
Waterfowl - goose	☐	________
Waterfowl - goose breeder	☐	________
Waterfowl - other domestic _________________	☐	________
Waterfowl - wild	☐	________
Wild bird - type: _______________________________	☐	________
Zoo bird - type: ___________________________	☐	________

CONTINUED > > > >

PART B1: INITIAL PREMISES VISIT REPORT - INITIAL ASSESSMENT AND SAMPLING

INVESTIGATION ID: ______________________________
Premises Identifier / Name: ______________________________

B1.9(4) ANIMAL TYPES (continued)

BOVINE:	Present on Premises?	Identify (√) MAIN type(s)
Dairy - mature cows	☐	________
Dairy - heifers	☐	________
Dairy - feeder calves (veal, steers)	☐	________
Dairy - bulls	☐	________
Beef - cow/calf	☐	________
Beef - backgrounder (on grass or growth ration)	☐	________
Beef - feeder - steers and/or heifers	☐	________
Beef - finishers - steers and/or heifers	☐	________
Beef - feeder / finisher - cull cows and/or bulls	☐	________
Beef - bulls	☐	________
Farmed bison - breeders	☐	________
Farmed bison - feeders	☐	________
Wild bison	☐	________
Other ______________________________	☐	________

SMALL RUMINANTS:		
Sheep - Breeding operation - ewes and lambs	☐	________
Sheep - Breeding and Feeder operation	☐	________
Sheep - Feeder operation only	☐	________
Sheep - Wool production	☐	________
Sheep - Milk production	☐	________
Rams	☐	________
Goats - Breeding operation - does and kids	☐	________
Goats - Breeding and Feeder operation	☐	________
Goats - Feeder operation only	☐	________
Goats - Hair production	☐	________
Goats - Milk production	☐	________
Bucks	☐	________
Llama / vicuna / alpaca - Breeding - female and cria	☐	________
Llama / vicuna / alpaca - Breeding & Feeding	☐	________
Llama / vicuna / alpaca - Feeder operation only	☐	________
Llama / vicuna / alpaca - Wool production	☐	________
Llama / vicuna / alpaca - Males	☐	________
Wild small ruminants - hunt preserve	☐	________
Other ______________________________	☐	________

CERVIDS:		
Captive white tailed deer	☐	________
Captive mule deer	☐	________
Captive fallow deer	☐	________
Captive red deer	☐	________
Captive reindeer / caribou	☐	________
Captive elk	☐	________
Captive red deer / elk crosses	☐	________
Other captive cervids ________________________	☐	________

CONTINUED > > > >

PART B1: INITIAL PREMISES VISIT REPORT - INITIAL ASSESSMENT AND SAMPLING

INVESTIGATION ID: ______________________________
Premises Identifier / Name: ______________________________

B1.9(4) ANIMAL TYPES (continued)

	Present on Premises?	Identify (√) MAIN type(s)
**EQUINE:**		
Horses - Breeding operation - mares and foals	☐	________
Horses - Stallions	☐	________
Horses - Standardbred Racing training facility	☐	________
Horses - Thoroughbred Racing training facility	☐	________
Horses - Quarter Horse Racing / Training facility	☐	________
Horses - Performance - endurance	☐	________
Horses - Performance - dressage	☐	________
Horses - Performance - rodeo	☐	________
Horses - Performance - chuckwagon	☐	________
Horses - Performance - other __________________	☐	________
Horses - Boarding facility (pleasure / performance)	☐	________
Horses - Draft	☐	________
Horses - Miniature	☐	________
Donkeys	☐	________
Mules	☐	________
Equine - food animal production	☐	________
Other ______________________	☐	________
**SWINE:**		
Gilts and Sows - Open and/or Bred	☐	________
Sows - Farrowing and Nursing	☐	________
Piglets - Nursing	☐	________
Weaners	☐	________
Grower / Finisher	☐	________
Boars	☐	________
Farmed Wild Swine	☐	________
Wild Swine (hunt preserve)	☐	________
Pets / Exotic species	☐	________
Other - ________________________________	☐	________
**OTHER MAMMALS:**		
Farmed mink	☐	________
Farmed foxes	☐	________
Farmed rabbits - fur	☐	________
Farmed rabbits - meat	☐	________
Farmed bees	☐	________
Other farmed animals ________________________	☐	________
Other wild species __________________________	☐	________
Dogs - pets	☐	________
Dogs - kennel / breeder	☐	________
Cats - pets	☐	________
Cats - kennel / breeder	☐	________
Other pet species __________________________	☐	________

PART B1: INITIAL PREMISES VISIT REPORT - INITIAL ASSESSMENT AND SAMPLING

INVESTIGATION ID: ______________________

Premises Identifier / Name: ______________________

B1.10 (1) SITE PLAN OF THE PREMISES **NOTE: Extra sheets are included at the end.**

Show **ALL** buildings on the premises. Mark **NORTH** on the Site Plan. **TAKE PHOTOGRAPHS!!!!!**

For those buildings which house animals: Use the extra sheets to draw buildings that are multi-level, or that are are partitioned off to hold different ages or categories of animals.

Draw yards and pasture areas that animals have access to, cropland, woodlots, entrance ways off public roads (mark GPS point with '**X**'), roads, railways, streams, ponds, lakes, public right of way or footpath, or other significant features. (Use the MAP for locating these, if it is easier.)

Outline the perimeter of the property. The size of the property is: _______________ acres / hectares (select one)

Is there ANY STANDING WATER on the property? **YES** _____ / **NO** _____

If YES: MARK this on the Site Plan. **DONE** ______

Do waterfowl and / or wildlife visit this standing water? **YES** _____ / **NO** _____

Locate WATER SOURCE on Site Plan; describe it (eg. well, municipal system):

Is the animals' drinking water / misting water treated (i.e. with chlorine)? **YES** _____ / **NO** _____

PART B1: INITIAL PREMISES VISIT REPORT - INITIAL ASSESSMENT AND SAMPLING

INVESTIGATION ID: ______________________

Premises Identifier / Name: ______________________

B1.10 (2) SITE PLAN KEY

Label and describe buildings (eg. B1 = barn 1; B1A = section A in barn 1; H2 = house 2; FM = feed mill, etc.)

LABEL	DESCRIPTION OF BUILDINGS / YARDS / PASTURES / ETC.

PART B1: INITIAL PREMISES VISIT REPORT - INITIAL ASSESSMENT AND SAMPLING

INVESTIGATION ID: ______________________________

Premises Identifier / Name: ______________________________

B1.11 CLINICAL HISTORY OF THE PREMISES

Identify the main person(s) responsible for the care of the animals:

*** * * INTERVIEW THE ABOVE INDIVIDUAL(S) FOR THE FOLLOWING SECTION * * ***

Have you noticed ANY signs of illness / decreased production / increased mortality / ill health in ANY of the animals in the past 28 days? **YES** _____ / **NO** _____

If YES: What DATE did you first notice these signs of ill health? (YY/MM/DD) _____ / _____ / _____

Starting with the date above, provide a CLEAR HISTORY, in order, of the signs of ill health in the animals.

**Date observed**	**In what animals?**				
**YY / MM / DD**	**TYPE**	**AGE**	**WHERE on the SITE PLAN were they located?**	**What signs of ill health were observed? Did these animals receive any treatments*?**	**WHERE on the SITE PLAN are they presently located?**

*** Treatments include any preventative or therapeutic products administered to these animals.**

INVESTIGATION ID: ______________________

Premises Identifier / Name: ______________________

B1.12 INVENTORY OF ANIMALS ON THIS PREMISES AND RECORD OF DIAGNOSTIC SAMPLE COLLECTION

List, describe thoroughly, and locate (based on the Site Plan of the Premises) **ALL** of the animals on this premises. (Use back of page if more space is required.) **Include comments on NON-FARMED animals also (i.e. feral cats / rodents / wildlife / wild birds / insects).**

NOTE: Treatments include both preventative (i.e. vaccines) and therapeutic agents administered to the animals.

PLEASE NOTE: IF NO RISK OF A REPORTABLE ANIMAL DISEASE IS IDENTIFIED, THEN NO SAMPLES NEED TO BE TAKEN!

Location on Site Plan	Animal Description:		Total here today	Initial Placement / Stocking Date (YY/MM/DD)	Total initially placed / stocked	Expected Movement / Slaughter Date (YY/MM/DD)	Are Clinical Signs Present? (Y/N) Have these animals received any TREATMENTS? (Y/N) *If YES to either* - Describe:	Were samples taken? (Y/N)	If the animals were sampled: Record the IDENTIFICATION NUMBERS and TYPES of samples HERE
	Type	Age							
								Y / N	
								Y / N	
								Y / N	
								Y / N	
								Y / N	

TAKE PHOTOGRAPHS!!!!!

INVESTIGATION ID: ______________________________

Premises Identifier / Name: ______________________________

B1.13 RESIDENTS ON THE PREMISES

Do people reside on the premises? **YES** _____ / **NO** _____

If YES: Provide complete information on all residents. (Use back of page if more space is required.)

Name and Contact Number	Living location (as per premises map)	Do they own the premises? If YES, indicate this. Do they work on the premises? If YES, describe duties.	List and give details of ANY OTHER CONTACT with susceptible species, aside from the barns / enclosures on this premises.

INVESTIGATION ID: ______________________

Premises Identifier / Name: ______________________

B1.14 EMPLOYEES OF THE PREMISES

Provide complete contact information for all employees of the premises, if they live off site.
(Use back of page if more space is required.)

Name, Address & Phone	Work schedule / duties	List and give details of ANY OTHER CONTACT with susceptible species, aside from the barns / enclosures on this premises.

INVESTIGATION ID: ______________________

Premises Identifier / Name: ______________________

B1.15 RISK ASSESSMENT

This premises MUST be considered as **'HIGH RISK'** if any of the following statements is **TRUE**:

This premises was identified as a **SUSPECT INFECTED** premises based on history and necropsy findings by a Species Specialist and/or a Diagnostic Laboratory.	**T / F**
This premises was identified as a **SUSPECT INFECTED** premises based on the identification of HIGH RISK animals during ante-mortem and/or post-mortem inspection by the Veterinarian-in-Charge at a registered establishment (abattoir), or the designated responsible person at a provincial abattoir.	**T / F**
This premises was identified as a **SUSPECT or PRESUMED INFECTED** premises based on the detection of the agent, or a serological response to the agent, by a non-CFIA Laboratory.	**T / F**
This premises was identified as a **SUSPECT or PRESUMED or CONFIRMED INFECTED** premises based on the detection of the agent, or a serological response to the agent, by a CFIA Laboratory.	**T / F**
This premises is considered to be **SUSPECT INFECTED** because animals are clinically ill, with signs consistent with a reportable disease, as assessed by the investigating veterinarian(s).	**T / F**
**FOLLOW UP INVESTIGATION:** This premises is a SIGNIFICANT Contact to a High Risk **SUSPECT or PRESUMED or CONFIRMED INFECTED** Premises (e.g. TRACE-IN, TRACE-OUT).	**T / F**
**FOLLOW UP INVESTIGATION:** This premises is located within 1 kilometre of a High Risk **SUSPECT** or **PRESUMED or CONFIRMED INFECTED** Premises.	**T / F**
**FOLLOW UP INVESTIGATION:** This premises is located within 1 to 3 kilometres of a High Risk **SUSPECT** or **PRESUMED or CONFIRMED INFECTED** Premises.	**T / F**
**FOLLOW UP INVESTIGATION:** This premises is located within 3 to 5 kilometres of a High Risk **SUSPECT** or **PRESUMED or CONFIRMED INFECTED** Premises.	**T / F**

IF NONE OF THE ABOVE ARE 'TRUE':

This premises SHOULD be considered as **'HIGH RISK'** if **SEVERAL** of the following statements are **TRUE**:

There are **domestic** waterfowl maintained on the premises.	**T / F**
There is evidence / history of subnormal health in any barn / group of animals on the premises. **(B1.11)**	**T / F**
Some animals have access to the outdoors.	**T / F**
There has been evidence of wildlife or wild birds on or around this premises over the past 90 days.	**T / F**
Wild life and/or wild birds are able to enter the barns.	**T / F**
The animals on this premises may have direct contact with animals from other premises (i.e. communal pasture) and/or with wild species.	**T / F**
This premises is part of a multi-site production system **(B1.9)**.	**T / F**
This premises is NOT a closed production unit **(B1.9)**.	**T / F**
This premises is at a HIGH RISK of becoming infected due to many movements onto / off of it.	**T / F**
Access to the barns is not restricted.	**T / F**
Outside footwear is worn inside the barns.	**T / F**
Residents and/or employees have contact with susceptible species elsewhere **(B1.13, B1.14).**	**T / F**
Water used for the animals (eg. drinking, misting) is not treated **(B1.10(1)).**	**T / F**
There is a history of foreign travel and/or products from foreign countries being brought on-site.	**T / F**
Record **any other Risk Factors** for this Disease that you have identified:<br>(Use back of page if necessary; attach Disease Specific Checklist)	**T / F**

INVESTIGATION ID: ______________________________

Premises Identifier / Name: ______________________________

B1.16 RISK CATEGORY DECISION

This premises is being assessed as:

- ☐ **HIGH RISK**
- ☐ **MEDIUM**
- ☐ **LOW RISK**
- ☐ **NO RISK**

B1.17 CONTROL OF THE PREMISES

Are CFIA controls being put in place on this premises? **YES** _____ / **NO** _____

If YES: Identify ALL reasons WHY the controls are being put in place:

- ☐ The premises is HIGH RISK.
- ☐ This is a Follow-up Investigation from a High Risk Suspect Premises.
- ☐ OTHER - Describe:

Record details on all Operational Activities that may be impacted by the Movement Controls.
(For example: products and / or animals going to market, replacement animals being brought in or moved out, within premises' movements.)

**Operational Activity**	**Expected Movement Schedule**

INVESTIGATION ID: ______________________
Premises Identifier / Name: ______________________

B1.18 ACTIONS BASED ON RISK CATEGORY

(1) If NO RISK: **END VISIT HERE... RETURN TO OFFICE; COMPLETE DATA ENTRY**

(2) If LOW RISK: Samples to be submitted to:

☐ NON-CFIA Laboratory — Laboratory Name / Location: ______________
Submission #: ______________

OR

☐ CFIA Laboratory — Laboratory Name / Location: ______________
LSTS Submission #: ______________

> > > > Did you contact the CFIA Laboratory? **YES** _____ / **NO** _____

☐ Controls (eg. signs) set up at entranceway, if required.

END VISIT HERE... RETURN TO OFFICE; COMPLETE DATA ENTRY & SAMPLE SUBMISSION.

(3) If HIGH or MEDIUM RISK:
Samples to be submitted to:

☐ NON-CFIA Laboratory — Laboratory Name / Location: ______________
Submission #: ______________

AND / OR

☐ CFIA Laboratory — Laboratory Name / Location: ______________
LSTS Submission #: ______________

> > > > Did you contact the CFIA Laboratory? **YES** _____ / **NO** _____

☐ Establish the Time Period for The Trace Investigations (B2.3) **NOT APPLICABLE** _____

The Critical Period (CP) for introduction of this disease is set at: __________ days.
Using this CP, counting back from the date of the **first evidence of disease***,
the starting date for the Tracing Period is: _____ / _____ / _____ (YY/MM/DD).
* **If required, consult with your Area Disease Specialist to determine this.**

☐ Add the above date to Sections B2.3 of Part B2 of the Investigation Questionnaire.

☐ Controls (eg. signs) set up at entranceway

☐ Call in Report of '**HIGH RISK**' to Area Disease Specialist and Inspection Manager.

☐ If required, ensure contact to the local Public Health Office is made. ☐ Handout provided ☐ Called

☐ Sign off on this Part (B1) of the questionnaire. If possible, make a copy of Part B1 to keep as a reference while filling out Part B2. Give the original Part B1, the samples and any used cameras to the Inspector to take back to District Office for data entry and sample submission. (NOTE: If there will be a long delay due to travel, consider either calling in, or faxing in the Part B1 information to Support Staff at the District Office for more rapid data entry into CEMRS.)

☐ Stay on farm (with phone and cameras) and complete Part B2 of the Investigation Questionnaire.

CONTACTS (Name & Telephone Number):
Veterinarian on site: ______________
Inspector at gate: ______________
Area Disease Specialist: ______________
Inspection Manager: ______________
Support Staff at District Office: ______________

INVESTIGATION ID: ____________________

Premises Identifier / Name: ____________________

EXTRA SHEETS FOR PART B1.10(1) SITE PLAN OF THE PREMISES

PART B1: INITIAL PREMISES VISIT REPORT - INITIAL ASSESSMENT AND SAMPLING

INVESTIGATION ID: ______________________

Premises Identifier / Name: ______________________

EXTRA SHEETS FOR PART B1.10 SITE PLAN OF THE PREMISES

